# Improved NMR transfer of magnetization from protons to half-integer spin quadrupolar nuclei at moderate and high magic-angle spinning frequencies

**DOI:** 10.5194/mr-2-447-2021

**Published:** 2021-06-17

**Authors:** Jennifer S. Gómez, Andrew G. M. Rankin, Julien Trébosc, Frédérique Pourpoint, Yu Tsutsumi, Hiroki Nagashima, Olivier Lafon, Jean-Paul Amoureux

**Affiliations:** 1 Univ. Lille, CNRS, Centrale Lille, Univ. Artois, UMR 8181 – UCCS – Unité de Catalyse et Chimie du Solide, Lille, 59000, France; 2 Univ. Lille, CNRS, INRAE, Centrale Lille, Univ. Artois, FR 2638 – IMEC – Fédération Chevreul, Lille, 59000, France; 3 Bruker Japan, 3-9 Moriya, Kanagawa, Yokohama, Kanagawa, 221-0022, Japan; 4 Interdisciplinary Research Center for Catalytic Chemistry, National Institute of Advanced Industrial Science and Technology (AIST), 1-1-1 Higashi, Tsukuba, Ibaraki, 305-8565, Japan; 5 Institut Universitaire de France, 1 rue Descartes, Paris, 75231, France; 6 Riken NMR Science and Development Division, Yokohama, Kanagawa, 230-0045, Japan; 7 Bruker Biospin, 34 rue de l'industrie, Wissembourg, 67166, France; a present address: Sorbonne Université, CNRS, Collège de France, Laboratoire de Chimie de la Matière Condensée de Paris (LCMCP), 4 place Jussieu, Paris, 75005, France

## Abstract

Half-integer spin quadrupolar nuclei are the only magnetic isotopes for the majority of the chemical elements. Therefore, the transfer of polarization from protons to these isotopes under magic-angle spinning (MAS) can provide precious insights into the interatomic proximities in hydrogen-containing solids, including organic, hybrid, nanostructured and biological solids. This transfer has recently been combined with dynamic nuclear polarization (DNP) in order to enhance the NMR signal of half-integer quadrupolar isotopes. However, the cross-polarization transfer lacks robustness in the case of quadrupolar nuclei, and we have recently introduced as an alternative technique a D -RINEPT (through-space refocused insensitive nuclei enhancement by polarization transfer) scheme combining a heteronuclear dipolar recoupling built from adiabatic pulses and a continuous-wave decoupling. This technique has been demonstrated at 9.4 T with moderate MAS frequencies, $\nu_{R}\approx 10$ –15 kHz, in order to transfer the DNP-enhanced ${}^{1}$ H polarization to quadrupolar nuclei. Nevertheless, polarization transfers from protons to quadrupolar nuclei are also required at higher MAS frequencies in order to improve the ${}^{1}$ H resolution. We investigate here how this transfer can be achieved at $\nu_{R}\approx 20$ and 60 kHz. We demonstrate that the D -RINEPT sequence using adiabatic pulses still produces efficient and robust transfers but requires large radio-frequency (rf) fields, which may not be compatible with the specifications of most MAS probes. As an alternative, we introduce robust and efficient variants of the D -RINEPT and PRESTO (phase-shifted recoupling effects a smooth transfer of order) sequences using symmetry-based recoupling schemes built from single and composite π pulses. Their performances are compared using the average Hamiltonian theory and experiments at $B_{0}=18.8$ T on γ -alumina and isopropylamine-templated microporous aluminophosphate (AlPO ${}_{4}$ -14), featuring low and significant ${}^{1}$ H– ${}^{1}$ H dipolar interactions, respectively. These experiments demonstrate that the ${}^{1}$ H magnetization can be efficiently transferred to ${}^{27}$ Al nuclei using D -RINEPT with $\mathrm{SR}4_{1}^{2}$ (270 ${}_{0}$ 90 ${}_{180}$ ) recoupling and using PRESTO with $R22_{2}^{7}$ (180 ${}_{0}$ ) or $R16_{7}^{6}$ (270 ${}_{0}$ 90 ${}_{180}$ ) schemes at $\nu_{R}=20$ or 62.5 kHz, respectively. The D -RINEPT and PRESTO recoupling schemes complement each other since the latter is affected by dipolar truncation, whereas the former is not.

We also analyze the losses during these recoupling schemes, and we show how these magnetization transfers can be used at $\nu_{R}=62.5$ kHz to acquire in 72 min 2D HETCOR (heteronuclear correlation) spectra between ${}^{1}$ H and quadrupolar nuclei, with a non-uniform sampling (NUS).

## 1 Introduction

Quadrupolar nuclei with a nuclear spin quantum number S=3/2 , 5/2 , 7/2 or 9/2 are the only NMR-active isotopes for over 60 % of the chemical elements of the first six periods of the periodic table, including six of the eight most abundant elements by mass in the Earth's crust: O, Al, Ca, Na, Mg and K (Ashbrook and Sneddon, 2014). A wide range of materials, including organic compounds, biological macromolecules, and nanostructured or hybrid materials, contain half-integer spin quadrupolar nuclei and protons. Proximities between these isotopes have notably been probed in solid-state NMR experiments by transferring the polarization of protons to half-integer quadrupolar nuclei through dipolar couplings under magic-angle spinning (MAS) conditions (Rocha et al., 1991; Hwang et al., 2004; Peng et al., 2007; Vogt et al., 2013; Chen et al., 2019). More recently, this polarization transfer has been combined under MAS with dynamic nuclear polarization (DNP) in order to enhance the NMR signals of half-integer spin quadrupolar nuclei (Vitzthum et al., 2012; Perras et al., 2015a; Nagashima et al., 2020). This approach has notably allowed for the detection of insensitive quadrupolar nuclei with low natural abundance, such as ${}^{17}$ O or ${}^{43}$ Ca, or low gyromagnetic ratio, γ , such as ${}^{47,49}$ Ti, ${}^{67}$ Zn or ${}^{95}$ Mo, near surfaces of materials (Perras et al., 2015a, 2016, 2017; Blanc et al., 2013; Hope et al., 2017; Lee et al., 2017; Nagashima et al., 2020, 2021; Li et al., 2018​​​​​​​).

This transfer has originally been achieved using cross-polarization under MAS (CPMAS) (Harris and Nesbitt, 1988). Nevertheless, this technique lacks robustness for quadrupolar nuclei since the spin-locking of the central transition (CT) between energy levels ±1/2 is sensitive to the strength of the quadrupole interaction, the offset, the chemical shift anisotropy (CSA) and the radio-frequency (rf) field inhomogeneity (Vega, 1992; Amoureux and Pruski, 2002; Tricot et al., 2011). Furthermore, CPMAS experiments require a careful adjustment of the rf field applied to the quadrupolar isotope in order to fulfill the Hartmann–Hahn conditions, $(S+1/2)\nu_{1S}+\varepsilon \nu_{1H}=n\nu_{R}$ , where $\nu_{1S}$ and $\nu_{1H}$ denote the amplitudes of the rf fields applied to the S quadrupolar isotope and to the protons, respectively; ε=±1 , n=±1 , or ±2 ; and $\nu_{R}$ denotes the MAS frequency, while avoiding the rotary resonance recoupling ( $R^{3}$ ) $\nu_{1S}=p\nu_{R}/(S+1/2)$ with p=0 , 1, 2 and 3 (Amoureux and Pruski, 2002; Ashbrook and Wimperis, 2009). Moreover, the magnetization of the quadrupolar nuclei cannot be spin-locked for some crystallite orientations, which leads to line-shape distortions (Barrie, 1993; Hayashi and Hayamizu, 1993; Ding and Mcdowell, 1995).

These issues have been circumvented by use of the PRESTO (phase-shifted recoupling effects a smooth transfer of order) scheme (Perras et al., 2015a, b; Zhao et al., 2004) and, more recently, the through-space refocused INEPT (denoted RINEPT hereafter) (Nagashima et al., 2020; Giovine et al., 2019). These schemes benefit from higher robustness than CPMAS since they do not employ a spin-lock on the quadrupolar channel but instead a limited number (two or three) of CT selective pulses. In these sequences, the dipolar interactions between protons and quadrupolar nucleus are reintroduced by applying on the ${}^{1}$ H channel symmetry-based recoupling sequences, such as $R18_{2}^{5}$ for PRESTO or $\mathrm{SR}4_{1}^{2}$ for RINEPT (Zhao et al., 2001; Brinkmann and Kentgens, 2006a). In the case of recoupling sequences built from single square π pulses, the RINEPT sequence using $\mathrm{SR}4_{1}^{2}$ (denoted RINEPT- $\mathrm{SR}4_{1}^{2}$ ) is more efficient than PRESTO at $\nu_{R}\geq 60$ kHz because of its higher robustness to rf field inhomogeneity and ${}^{1}$ H offset and CSA. At $\nu_{R}<20$ kHz, the PRESTO technique is more efficient since the efficiency of RINEPT- $\mathrm{SR}4_{1}^{2}$ is reduced by the increased losses due to ${}^{1}$ H– ${}^{1}$ H interactions during the $\mathrm{SR}4_{1}^{2}$ recoupling and the windows used to rotor synchronize the $\mathrm{SR}4_{1}^{2}$ blocks, whereas the PRESTO sequence is devoid of these windows (Giovine et al., 2019).

Recently, we have introduced a novel variant of the RINEPT sequence by employing the $\mathrm{SR}4_{1}^{2}$ recoupling built (i) from tanh/tan (tt) adiabatic inversion pulses, (ii) continuous-wave (CW) irradiations during the windows, and (iii) composite π/2 and π pulses on the ${}^{1}$ H channel, in order to limit the losses due to ${}^{1}$ H– ${}^{1}$ H interactions and improve the transfer efficiency at moderate MAS frequencies (Nagashima et al., 2020, 2021). This novel RINEPT variant, denoted RINEPT-CWc- $\mathrm{SR}4_{1}^{2}(\mathrm{tt})$ , is more efficient than PRESTO and CPMAS at $\nu_{R}\approx 12.5$ kHz, and it has been combined with DNP to detect the NMR signals of quadrupolar nuclei with small dipolar coupling with protons, including the low- γ isotopes, such as ${}^{47,49}$ Ti, ${}^{67}$ Zn or ${}^{95}$ Mo, and unprotonated ${}^{17}$ O nuclei. Furthermore, for quadrupolar nuclei subject to large dipolar interactions, such as ${}^{17}$ O nuclei of OH groups, we have shown that a RINEPT-CWc- $\mathrm{SR}4_{1}^{2}(\mathrm{tt})$ version with only two pulses on the quadrupolar channel is more efficient that its PRESTO counterpart (Nagashima et al., 2021).

However, several NMR experiments require the transfer of ${}^{1}$ H magnetization to quadrupolar nuclei at $\nu_{R}>12.5$ kHz. In particular, MAS frequencies of $\nu_{R}\geq 20$ kHz are needed to avoid the overlap between the center bands and the spinning sidebands of satellite transitions (STs) in ${}^{27}$ Al NMR spectra at 18.8 T. In addition, magnetization transfers at $\nu_{R}\geq 60$ kHz are advantageous to acquire through-space heteronuclear correlation ( D -HETCOR) 2D spectra between protons and quadrupolar nuclei endowed with high resolution along the ${}^{1}$ H dimension since fast MAS averages out the ${}^{1}$ H– ${}^{1}$ H dipolar couplings.

Concurrently, we have demonstrated that the efficiency of PRESTO transfers using the $R16_{7}^{6}$ recoupling can be improved at $\nu_{R}=62.5$ kHz using (270 ${}_{0}$ 90 ${}_{180}$ ) composite π pulses as a basic inversion element, where the standard notation for the pulses is used: $\xi_{\phi }$ denotes a rectangular, resonant rf pulse with flip angle ξ and phase ϕ in degrees (Giovine et al., 2019). More recently, $\mathrm{SR}4_{1}^{2}$ and $R12_{3}^{5}$ recoupling schemes built from (90 ${}_{-45}$ 90 ${}_{45}$ 90 ${}_{-45}$ ) composite π pulses have been proposed, but they have not yet been incorporated into RINEPT transfers (Perras et al., 2019). Globally, no systematic study of the $RN_{n}^{\nu }$ ​​​​​​​ recouplings built from composite π pulses has been carried out.

In the present article, we investigate the use of RINEPT-CWc using an adiabatic recoupling scheme at the higher MAS frequencies of $\nu_{R}=20$ and 62.5 kHz. We demonstrate using numerical simulations of spin dynamics and experiments on γ -alumina and isopropylamine-templated microporous aluminophosphate (AlPO ${}_{4}$ -14) (hereafter AlPO ${}_{4}$ -14) that the rf requirement of this technique increases with the ${}^{1}$ H– ${}^{1}$ H dipolar interactions. In practice, this rf requirement is not compatible with the specifications of most MAS probes at $\nu_{R}\geq 20$ kHz, even for moderate ${}^{1}$ H– ${}^{1}$ H dipolar interactions. As an alternative, we introduce variants of the PRESTO and RINEPT sequences by selecting with average Hamiltonian (AH) theory recoupling schemes built from single rectangular or composite π pulses. Finally, using experiments on γ -alumina and AlPO ${}_{4}$ -14, which feature small and moderate ${}^{1}$ H– ${}^{1}$ H dipolar interactions, respectively, we identify the most robust and efficient PRESTO and RINEPT transfers at $B_{0}=18.8$ T with $\nu_{R}=20$ and 62.5 kHz.

## 2 Pulse sequences and theory

### 2.1 PRESTO

#### 2.1.1 Single-quantum heteronuclear dipolar recoupling

A $RN_{n}^{\nu }$ sequence, where N is an even positive integer and n and ν are integers, consists of N/2 pairs of elements $R_{\phi }R_{-\phi }^{\prime }$ ​​​​​​​, with ϕ=πν/N radians an overall phase shift. $R_{\phi }$ is an inversion pulse with a duration of $nT_{R}/N$ , where $T_{R}=1/\nu_{R}$ is the rotor period, and $R_{-\phi }^{\prime }$ is an inversion pulse derived from R by changing the sign of all phases. R and $R^{\prime }$ are identical when they are amplitude modulated; i.e., all phase shifts are multiples of π . The rf field requirement of $RN_{n}^{\nu }$ is equal to

1 $$\nu_{1}=\frac{N}{n}\frac{\xi^{\mathrm{tot}}}{2\pi }\nu_{R},$$

where $\xi^{\mathrm{tot}}=\sum_{i=1}^{P}\xi^{i}$ is the sum of the flip angles of the P individual pulses of the R element.

**Figure 1:**
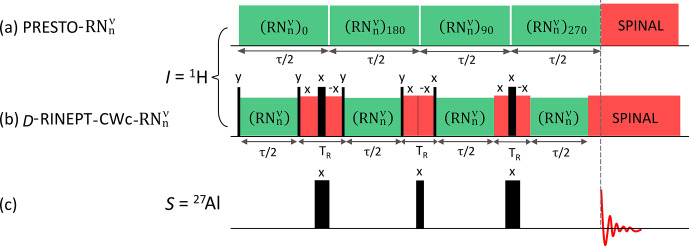
The ${}^{1}$ H → ${}^{27}$ Al **(a, c)** PRESTO- $RN_{n}^{\nu }$ and **(b, c)** D -RINEPT-CWc- $RN_{n}^{\nu }$ pulse sequences. Those applied to the ${}^{1}$ H channel are displayed in panels **(a)** and **(b)**, whereas that applied to the ${}^{27}$ Al channel is shown in panel **(c)**. The narrow and broad black bars represent π/2 and π pulses, respectively. The acquisition of the free-induction decays (FIDs) (indicated with the vertical dashed line) starts after **(a)** the end of the $RN_{n}^{\nu }$ block in the case of PRESTO or **(b)** on top of the echo shifted with $T_{R}/2$ with respect to the end of the last recoupling block in the case of RINEPT.

In the PRESTO sequence (Fig. 1a), symmetry-based γ -encoded $RN_{n}^{\nu }$ schemes applied to the ${}^{1}$ H channel reintroduce the $\left|m\right|=2$ space components and the single-quantum (SQ) terms of the heteronuclear dipolar couplings between the protons and the quadrupolar nuclei, as well as the ${}^{1}$ H CSA, while they suppress the contributions of ${}^{1}$ H isotropic chemical shifts, the heteronuclear J couplings with protons, and the ${}^{1}$ H– ${}^{1}$ H dipolar couplings to the first-order AH (Zhao et al., 2004). The heteronuclear dipolar interaction is characterized by a space rank l and a spin rank λ . A γ -encoded $\left|m\right|=2$ SQ heteronuclear dipolar recoupling must selectively reintroduce the two components {l,m,λ,μ}={2,2,1,μ} and {2,-2,1,-μ} of the heteronuclear dipolar coupling and ${}^{1}$ H CSA with μ=±1 , while all other components must be suppressed.

During these recoupling schemes, the contribution of the dipolar coupling between $I{=}^{1}$ H and S nuclei to the first-order Hamiltonian is equal to (Zhao et al., 2004)

2 $${\bar{H}}_{D,IS}^{\left(1\right)}=\omega_{D,IS}S_{z}\left[I^{+}\mathrm{exp}\left(i2\varphi \right)+I^{-}\mathrm{exp}\left(-i2\varphi \right)\right],$$

where $I^{\pm }=I_{x}\pm iI_{y}$ symbols represent the shift operators, and the magnitude and phase of the recoupled I – S dipolar coupling are given by

3 $$\omega_{D,IS}=-\kappa \frac{\sqrt{3}}{2}b_{IS}{\mathrm{sin}}^{2}\left(\beta_{PR}^{D,IS}\right)$$

and

4 $$\varphi =\gamma_{PR}^{D,IS}-\omega_{R}t^{0},$$

respectively, where $b_{IS}$ is the dipolar coupling constant in rad/s, and κ is the scaling factor of the recoupled heteronuclear dipolar interaction, which depends on the $RN_{n}^{\nu }$ symmetry and the R element. The Euler angles $\left\{0,\beta_{PR}^{D,IS},\gamma_{PR}^{D,IS}\right\}$ relate the I – S ​​​​​​​ vector to the MAS rotor frame, and $t^{0}$ refers to the starting time of the recoupling. The norm of ${\bar{H}}_{D,IS}^{\left(1\right)}$ does not depend on the $\gamma_{PR}^{D,IS}$ angle, since these recoupling schemes are γ encoded (Pileio et al., 2007; Martineau et al., 2012). The Hamiltonian of Eq. (2) does not commute among different spin pairs; hence, the PRESTO sequence is affected by dipolar truncation; i.e., the transfer to distant nuclei is attenuated by the stronger couplings with nearby spins (Bayro et al., 2009).

As mentioned above, the SQ heteronuclear dipolar recoupling schemes also reintroduce the ${}^{1}$ H CSA with the same scaling factor κ but without commuting with the recoupled ${}^{1}$ H– S dipolar interactions. Therefore, in the case of large ${}^{1}$ H CSA, for instance at high magnetic fields, this interaction can interfere with the ${}^{1}$ H– S dipolar couplings, especially with the small ones. These interferences can be limited by the use of the PRESTO-III variant, depicted in Fig. 1a, c (Zhao et al., 2004), in which three CT-selective pulses are applied to the S channel. Indeed, the CT-selective π pulses partly refocus the ${}^{1}$ H CSA, which limits these interferences.

#### 2.1.2 Selection of the recoupling sequence

On the basis of the AH and spin dynamics simulations, the $R18_{1}^{7}$ and $R18_{2}^{5}$ schemes built from single rectangular π pulses were selected for heteronuclear dipolar recoupling at moderate MAS frequencies, $\nu_{R}$ ≈10 kHz (Zhao et al., 2001), while, more recently, sequences based on symmetries $R12_{5}^{4}$ , $R14_{6}^{5}$ , $R16_{7}^{6}$ , $R14_{8}^{5}$ , $R18_{8}^{7}$ , $R16_{9}^{6}$ , $R20_{9}^{8}$ and $R18_{10}^{7}$ using (270 ${}_{0}$ 90 ${}_{180}$ ) as inversion element were chosen for the measurement of ${}^{1}$ H CSA at fast MAS frequencies, $\nu_{R}\approx 60$ –70 kHz (Pandey et al., 2015). We also transferred the proton polarization to ${}^{27}$ Al nuclei at $\nu_{R}=62.5$ kHz using PRESTO with $R16_{3}^{2}$ recoupling built from a single rectangular π pulse (Giovine et al., 2019).

We screened here the $RN_{n}^{\nu }$ schemes built from single rectangular and composite π pulses to achieve γ -encoded $\left|m\right|=2$ heteronuclear SQ dipolar recoupling at $\nu_{R}=20$ or 62.5 kHz. Dipolar recoupling at $\nu_{R}\geq 60$ kHz is useful to correlate the signals of quadrupolar nuclei with high-resolution ${}^{1}$ H spectra without using homonuclear dipolar decoupling. We tested the three following composite π pulses: (1) (270 ${}_{0}$ 90 ${}_{180}$ ), which is offset compensated and amplitude modulated and has been employed in several $RN_{n}^{\nu }$ sequences (Giovine et al., 2019; Carravetta et al., 2000; Levitt, 2002; Pandey et al., 2015); (2) (90 ${}_{0}$ 240 ${}_{90}$ 90 ${}_{0}$ ), which compensates both rf inhomogeneity and offset (Freeman et al., 1980; Duong et al., 2019); and (3) (90 ${}_{-45}$ 90 ${}_{45}$ 90 ${}_{-45}$ ), which has homonuclear decoupling properties (Madhu et al., 2001). Adiabatic pulses cannot be employed for SQ heteronuclear dipolar recoupling since they yield vanishing scaling factors for the rotational components with μ≠0 (Nagashima et al., 2018).

A total of 109 $RN_{n}^{\nu }$ symmetries with 2≤N≤30 , 2≤n≤7 and 1≤ν≤11 were found which recouple the {2,±2,1,±1} or {2,∓2,1,±1} rotational components of the ${}^{1}$ H– S dipolar coupling and ${}^{1}$ H CSA. We selected the $RN_{n}^{\nu }$ recouplings based on those symmetries with rf field limited to $\nu_{1}\leq 120$ and 190 kHz for $\nu_{R}=20$ and 62.5 kHz, respectively. We only considered the $RN_{n}^{\nu }$ symmetries with 45≤ϕ≤135 ${}^{\circ }$ since sequences with ϕ close to 90 ${}^{\circ }$ are better compensated for rf field errors and inhomogeneities (Brinkmann and Kentgens, 2006b). The scaling factor, κ , of the recoupled ${}^{1}$ H– S dipolar interaction was calculated using the “C and R symmetries” Mathematica package (Carravetta et al., 2000; Brinkmann and Levitt, 2001; Brinkmann et al., 2000; Brinkmann and Edén, 2004).

These $RN_{n}^{\nu }$ symmetries eliminate the contribution of ${}^{1}$ H– ${}^{1}$ H dipolar interactions to the first-order Hamiltonian but not their contribution to the second order. The cross terms between ${}^{1}$ H– ${}^{1}$ H interactions in the second-order Hamiltonian can be written (Brinkmann and Edén, 2004):

5 $$\begin{array}{rl} {\bar{H}}^{(2),{\mathrm{DD}}_{1}\times {\mathrm{DD}}_{2}} & =\frac{1}{\nu_{R}}\sum_{\left\{1,2\right\}}\kappa_{\left\{1,2\right\}}^{{\mathrm{DD}}_{1}\times {\mathrm{DD}}_{2}}{\left[A_{l_{2}m_{2}}^{{\mathrm{DD}}_{2}}\right]}^{R}{\left[A_{l_{1}m_{1}}^{{\mathrm{DD}}_{1}}\right]}^{R}\\ & \times \mathrm{exp}\left[i\left(m_{1}+m_{2}\right)\omega_{R}t^{0}\right]\left[T_{\lambda_{2}\mu_{2}}^{{\mathrm{DD}}_{2}},T_{\lambda_{1}\mu_{1}}^{{\mathrm{DD}}_{1}}\right], \end{array}$$

where the sum is taken over all second-order cross terms {1,2} between the $\left\{l_{1},m_{1},\lambda_{1},\mu_{1}\right\}$ and $\left\{l_{2},m_{2},\lambda_{2},\mu_{2}\right\}$ rotational components of DD ${}_{1}$ and DD ${}_{2}$ ${}^{1}$ H– ${}^{1}$ H dipolar interactions, respectively. $\kappa_{\left\{1,2\right\}}^{{\mathrm{DD}}_{1}\times {\mathrm{DD}}_{2}}$ is the scaling factor of this cross term; ${\left[A_{l_{i}m_{i}}^{{\mathrm{DD}}_{i}}\right]}^{R}$ and $T_{\lambda_{i}\mu_{i}}^{{\mathrm{DD}}_{i}}$ denote the component $m_{i}$ of the $l_{i}$ th rank spatial irreducible spherical tensor $A^{{\mathrm{DD}}_{i}}$ in the MAS rotor-fixed frame and the component $\mu_{i}$ of the $\lambda_{i}$ th rank spin irreducible spherical tensor operator $T^{{\mathrm{DD}}_{i}}$ . Equation (5) indicates that the amplitude of the second-order Hamiltonian decreases at higher MAS frequencies. The magnitude of the cross terms between ${}^{1}$ H– ${}^{1}$ H interactions was estimated by calculating the Euclidean norm (Hu et al., 2009; Gansmüller et al., 2013):

6 $${∥\kappa_{\left\{1,2\right\}}^{{\mathrm{DD}}_{1}\times {\mathrm{DD}}_{2}}∥}_{2}=\sqrt{\sum_{\left\{1,2\right\}}{\left|\kappa_{\left\{1,2\right\}}^{{\mathrm{DD}}_{1}\times {\mathrm{DD}}_{2}}\right|}^{2}}.$$

For each basic element R , we selected the $RN_{n}^{\nu }$ schemes with the highest ratio $\kappa /{∥\kappa_{\left\{1,2\right\}}^{{\mathrm{DD}}_{1}\times {\mathrm{DD}}_{2}}∥}_{2}$ in order to minimize the interference of ${}^{1}$ H– ${}^{1}$ H dipolar interactions with the ${}^{1}$ H– S dipolar recoupling. Besides ${}^{1}$ H– ${}^{1}$ H dipolar interactions, other cross terms involving ${}^{1}$ H CSA and offset can also interfere with the ${}^{1}$ H– S dipolar recoupling. These cross terms can be expressed by Eq. (5), in which DD ${}_{1}$ and DD ${}_{2}$ indexes are substituted by other interactions, such as ${}^{1}$ H CSA or isotropic chemical shift ( $\delta_{\mathrm{iso}}$ ). For the selected symmetries, we estimated the magnitude of the cross terms between ${}^{1}$ H CSA or offset by calculating the Euclidean norms ${∥\kappa_{\left\{1,2\right\}}^{\mathrm{CSA}\times \mathrm{CSA}}∥}_{2}$ and ${∥\kappa_{\left\{1,2\right\}}^{\delta \mathrm{iso}\times \delta \mathrm{iso}}∥}_{2}$ given by Eq. (6).

The corresponding selected $RN_{n}^{\nu }$ sequences are listed in Tables S1 and S2 in the Supplement for $\nu_{R}=20$ and 62.5 kHz, respectively.

For $\nu_{R}=20$ kHz, according to the AH, the $RN_{n}^{\nu }$ sequence with the highest robustness to ${}^{1}$ H– ${}^{1}$ H dipolar interactions is $R22_{2}^{7}(180_{0}$ ). However, this recoupling is slightly less robust to ${}^{1}$ H CSA and offset than $R18_{2}^{5}(180_{0}$ ), which has already been reported. For this MAS frequency, the $RN_{n}^{\nu }$ schemes using the chosen composite pulses either required rf fields greater than 120 kHz, e.g., $\nu_{1}=130$ and 173 kHz for the R26 ${}_{3}^{7}$ schemes built from (90 ${}_{-45}$ 90 ${}_{45}$ 90 ${}_{-45}$ ) and (270 ${}_{0}$ 90 ${}_{180}$ ) pulses, or did not suppress efficiently the second-order cross terms between ${}^{1}$ H– ${}^{1}$ H interactions because of small rf field ( $\nu_{1}\leq 62.5$ kHz).

For $\nu_{R}=62.5$ kHz, the $RN_{n}^{\nu }$ sequences using composite π pulses recouple the ${}^{1}$ H– S dipolar interaction with a higher scaling factor than those built from single π pulses. According to AH, the (90 ${}_{0}$ 240 ${}_{90}$ 90 ${}_{0}$ ) basic element leads to the highest robustness to ${}^{1}$ H– ${}^{1}$ H interferences. Even if the amplitude of the cross terms is inversely proportional to the MAS frequency (Eq. 5), the amplitude of these terms is lower at $\nu_{R}=20$ than 62.5 kHz. The (270 ${}_{0}$ 90 ${}_{180}$ ) element is less robust to ${}^{1}$ H– ${}^{1}$ H interferences but benefits from a high robustness to offset. The selected $RN_{n}^{\nu }$ symmetries for this element include $R14_{6}^{5}$ and $R16_{7}^{6}$ , which have already been employed for the measurement of ${}^{1}$ H CSA and the transfer of ${}^{1}$ H polarization to half-integer quadrupolar nuclei at $\nu_{R}\geq 60$ kHz (Giovine et al., 2019; Pandey et al., 2015). The scaling factors κ of the ${}^{1}$ H– S dipolar interaction of the $RN_{n}^{\nu }$ schemes built from single π pulses are small with 45≤ϕ≤135 ${}^{\circ }$ ; hence, we also selected in Table S3 those with an extended ϕ range of 20–160 ${}^{\circ }$ . These recoupling schemes are less robust to offset than the $RN_{n}^{\nu }$ schemes built from (270 ${}_{0}$ 90 ${}_{180}$ ) element.

### 2.2 D -RINEPT

#### 2.2.1 Zero-quantum heteronuclear dipolar recoupling

In the D -RINEPT sequence, the ${}^{1}$ H– S dipolar interactions are reintroduced under MAS by applying non- γ -encoded two-spin order dipolar recoupling to the ${}^{1}$ H channel. These schemes reintroduce the $\left|m\right|=2$ space components and the zero-quantum (0Q) terms of the ${}^{1}$ H– S dipolar interaction and ${}^{1}$ H CSA; i.e., the rotational components {l,m,λ,μ}={2,±2,1,0} , while they suppress the contributions of ${}^{1}$ H isotropic chemical shifts, the heteronuclear J couplings with protons, and the ${}^{1}$ H– ${}^{1}$ H dipolar couplings to the first-order AH (Brinkmann and Kentgens, 2006a, b). The contribution of the ${}^{1}$ H– S dipolar coupling to this Hamiltonian is equal to (Giovine et al., 2019; Brinkmann and Kentgens, 2006a; Lu et al., 2012)

7 $${\bar{H}}_{D,IS}^{\left(1\right)}=2\omega_{D,IS}I_{z}S_{z},$$

where

8 $$\omega_{D,IS}=\kappa b_{IS}{\mathrm{sin}}^{2}\left(\beta_{PR}^{D,IS}\right)\mathrm{cos}\left(2\varphi \right).$$

The norm of ${\bar{H}}_{D,IS}^{\left(1\right)}$ depends on the φ phase, given by Eq. (4), and hence on the $\gamma_{PR}^{D,IS}$ angle. Therefore, these two-spin order dipolar recoupling schemes are non- γ -encoded. The Hamiltonian of Eq. (7) commutes among different spin pairs; hence, these recoupling schemes are not affected by dipolar truncation. Similarly, the recoupled ${}^{1}$ H CSA contribution to the first-order Hamiltonian is proportional to $I_{z}$ and hence also commutes with the recoupled ${}^{1}$ H– S dipolar interactions and does not interfere with the heteronuclear dipolar recoupling.

#### 2.2.2 Selection of the recoupling sequence

Different $RN_{n}^{\nu }$ sequences have been proposed to achieve non- γ -encoded $\left|m\right|=2$ two-spin order dipolar recoupling, including (i) symmetries $R(4n{)}_{n}^{2n-1}=R12_{3}^{5}$ , $R16_{4}^{7}$ , $R20_{5}^{9}$ , $R24_{6}^{11}$ , $R28_{7}^{13}$ and $R32_{8}^{15}$ for n=3 , 4, 5, 6, 7 and 8 using single π pulses as basic element, which have been employed to measure ${}^{1}$ H– ${}^{17}$ O dipolar couplings at $\nu_{R}=50$ kHz (Brinkmann and Kentgens, 2006b); (ii) $\mathrm{SR}4_{1}^{2}$ recoupling built from a single π pulse, which corresponds to the ${\left[R4_{1}^{2}R4_{1}^{-2}\right]}_{0}{\left[R4_{1}^{2}R4_{1}^{-2}\right]}_{120}{\left[R4_{1}^{2}R4_{1}^{-2}\right]}_{240}$ sequence and has been employed in the RINEPT scheme (Nagashima et al., 2021; Giovine et al., 2019); (iii) $R12_{3}^{5}$ and $\mathrm{SR}4_{1}^{2}$ schemes using a (90 ${}_{-45}$ 90 ${}_{45}$ 90 ${}_{-45}$ ) composite π pulse as a basic element, which have been incorporated into D -HMQC (heteronuclear multiple quantum coherence) at $\nu_{R}=36$ kHz (Perras et al., 2019), and (iv) $\mathrm{SR}4_{1}^{2}$ schemes built from a (tt) adiabatic pulse, which have been used in the RINEPT sequence (Nagashima et al., 2021, 2020). During the (tt) pulse, the instantaneous rf amplitude is equal to

9 $$\begin{array}{rl} & \omega_{1}(t)=\\ & \omega_{1,max}\left\{\begin{array}{ll} \mathrm{tanh}\left[\frac{8\xi t}{T_{R}}\right] & 0\leq t<T_{R}/8,\\ \mathrm{tanh}\left[2\xi \left(1-\frac{4t}{T_{R}}\right)\right] & T_{R}/8\leq t<T_{R}/4, \end{array}\right. \end{array}$$

where $\omega_{1,max}$ is the peak amplitude of the rf field, t refers to the time since the start of the pulse, which lasts $T_{R}/4$ when incorporated into the $\mathrm{SR}4_{1}^{2}$ recoupling scheme. The parameter ξ determines the rise and fall times of the pulse. Hence, in the frequency-modulated (FM) frame (Garwood and DelaBarre, 2001), the frequency offset is

10 $$\phi_{I}(t)=\frac{\Delta \nu_{0,max}}{2\theta \mathrm{tan}(\theta )}\mathrm{ln}\left\{\mathrm{cos}\left[\theta \left(1-8\frac{t}{T_{R}}\right)\right]\right\},$$

where $\Delta \nu_{0,max}$ is the peak amplitude of the carrier frequency modulation, and θ determines the frequency sweep rate in the center of the pulse. Here, we employed ξ=10 and $\theta =87{}^{\circ }=\mathrm{atan}(20)$ (Kervern et al., 2007; Nagashima et al., 2018, 2020).

We screened here the $RN_{n}^{\nu }$ schemes built from (180 ${}_{0}$ ), (270 ${}_{0}$ 90 ${}_{180}$ ), (90 ${}_{0}$ 240 ${}_{90}$ 90 ${}_{0}$ ) and (90 ${}_{-45}$ 90 ${}_{45}$ 90 ${}_{-45}$ ) inversion elements. A total of 58 $RN_{n}^{\nu }$ symmetries with 2≤N≤30 , 2≤n≤7 and 1≤ν≤11 were found which recouple the {2,±2,1,0} rotational components of the ${}^{1}$ H– S dipolar coupling and ${}^{1}$ H CSA. We only considered the $RN_{n}^{\nu }$ symmetries with 60≤ϕ≤120 ${}^{\circ }$ since the currently employed non- γ -encoded $\left|m\right|=2$ two-spin order heteronuclear dipolar recoupling schemes have 75≤ϕ≤90 ${}^{\circ }$ .

We calculated the scaling factor of the recoupled ${}^{1}$ H– S dipolar interaction and the Euclidean norm ${∥\kappa_{\left\{1,2\right\}}^{{\mathrm{DD}}_{1}\times {\mathrm{DD}}_{2}}∥}_{2}$ of the cross terms between ${}^{1}$ H– ${}^{1}$ H interactions using the “C and R symmetries” Mathematica package. For each basic element R , we selected the $RN_{n}^{\nu }$ schemes with the highest ratios $\kappa /{∥\kappa_{\left\{1,2\right\}}^{{\mathrm{DD}}_{1}\times {\mathrm{DD}}_{2}}∥}_{2}$ . The selected $RN_{n}^{\nu }$ sequences are listed in Table S4, along with the parameters of the $\mathrm{SR}4_{1}^{2}$ schemes built from the different basic elements R for the sake comparison. For these sequences, we calculated the Euclidean norms, ${∥\kappa_{\left\{1,2\right\}}^{\mathrm{CSA}\times \mathrm{CSA}}∥}_{2}$ and ${∥\kappa_{\left\{1,2\right\}}^{\delta \mathrm{iso}\times \delta \mathrm{iso}}∥}_{2}$ , in order to estimate the magnitudes of the cross terms between ${}^{1}$ H CSA and offset.

According to the AH, the (90 ${}_{0}$ 240 ${}_{90}$ 90 ${}_{0}$ ) composite π pulse yields the highest robustness to ${}^{1}$ H– ${}^{1}$ H dipolar interactions. However, the rf field requirement of the $RN_{n}^{\nu }$ sequences built from this composite pulse, $\nu_{1}=1.16N\nu_{R}/n$ , is not compatible at $\nu_{R}=62.5$ kHz with most 1.3 mm MAS probes (e.g., $\nu_{1}=291$ kHz for $\mathrm{SR}4_{1}^{2}$ ). Furthermore, the highest robustness to ${}^{1}$ H CSA and offset are achieved using the (270 ${}_{0}$ 90 ${}_{180}$ ) composite π pulse. The $\mathrm{SR}4_{1}^{2}$ schemes benefit from the highest robustness to ${}^{1}$ H CSA because of the three-step multiple-quantum super-cycle (Brinkmann and Edén, 2004; Brinkmann and Kentgens, 2006a). Contrary to the $RN_{n}^{\nu }$ with $\left|m\right|=2$ SQ heteronuclear dipolar recouplings, the rf field of the $RN_{n}^{\nu }$ with $\left|m\right|=2$ two-spin order schemes is always higher than 2 $\nu_{R}$ since these symmetries with 2n>N , such as $R12_{9}^{5}$ , have smaller κ scaling factors for the basic elements employed here.

In the case of the adiabatic $RN_{n}^{\nu }$ (tt) sequences, the determination of the scaling factors of the first- and second-order terms of the effective Hamiltonian is more cumbersome since they depend on the $\nu_{1,max}$ , $\Delta \nu_{0,max}$ , ξ and θ parameters (Nagashima et al., 2018). For example, the scaling factor of the $R12_{3}^{5}$ and $\mathrm{SR}4_{1}^{2}$ schemes is κ=0.31 for $\nu_{1,max}/\Delta \nu_{0,max}=0.685$ , ξ=10 and θ=87 ${}^{\circ }$ , and this value monotonously decreases for increasing $\nu_{1,max}/\Delta \nu_{0,max}$ ratios.

#### 2.2.3 D -RINEPT-CWc sequence

The D -RINEPT-CWc sequence is displayed in Fig. 1b and c. The ${}^{1}$ H– S dipolar couplings are reintroduced by applying the $RN_{n}^{\nu }$ schemes listed in Table S4 during the defocusing and refocusing delays τ , which are identical in this article, even if distinct delays can improve the transfer efficiency (Nagashima et al., 2021). As the two-spin order recoupling schemes are non- γ -encoded, they must be rotor synchronized. We used here a delay of $T_{R}$ between two successive $RN_{n}^{\nu }$ blocks. In the D -RINEPT-CWc sequence, a CW irradiation is applied during these delays in order to limit the losses due to ${}^{1}$ H– ${}^{1}$ H dipolar interactions (Nagashima et al., 2021). The nutation during this CW irradiation is eliminated by employing CW irradiations with opposite phases. Furthermore, the robustness to ${}^{1}$ H rf field inhomogeneity is improved by replacing the first π and second π/2 pulses by composite (90 ${}_{0}$ 180 ${}_{90}$ 90 ${}_{0}$ ) and (90 ${}_{90}$ 90 ${}_{0}$ ) pulses, respectively, with the CW irradiation being applied between the individual pulses (Freeman et al., 1980; Levitt and Freeman, 1979).

## 3 Numerical simulations

### 3.1 Simulation parameters

All simulations were performed using version 4.1.1 of the SIMPSON package (Bak et al., 2000). The powder average was performed using 462 $\left\{\alpha_{\mathrm{MR}},\beta_{\mathrm{MR}},\gamma_{\mathrm{MR}}\right\}$ Euler angles relating the molecular and rotor frames. This set of angles was obtained by considering 66 $\left\{\alpha_{\mathrm{MR}},\beta_{\mathrm{MR}}\right\}$ pairs and 7 $\gamma_{\mathrm{MR}}$ angles. The $\left\{\alpha_{\mathrm{MR}},\beta_{\mathrm{MR}}\right\}$ values were selected according to the REPULSION algorithm (Bak and Nielsen, 1997), while the $\gamma_{\mathrm{MR}}$ angles were regularly stepped from 0 to 360 ${}^{\circ }$ .

To accelerate the simulations, we used a ${}^{1}$ H → ${}^{15}$ N RINEPT transfer instead of the ${}^{1}$ H → ${}^{27}$ Al one, because the computing time is proportional to the cube of the size of the density matrix. Furthermore, in RINEPT experiments, only CT-selective pulses are applied to the quadrupolar nuclei; hence, the contribution of the STs to the signal can be disregarded. The ${}^{1}$ H → ${}^{15}$ N RINEPT transfer was simulated for a ${}^{15}$ N ${}^{1}$ H ${}_{4}$ spin system. A similar approach has already been applied for the simulation of the RINEPT transfer from protons to quadrupolar nuclei (Nagashima et al., 2021; Giovine et al., 2019). This ${}^{15}$ N ${}^{1}$ H ${}_{4}$ spin system comprises a tetrahedron of four protons with a ${}^{15}$ N nucleus on one of its symmetry axes. The dipolar coupling constants between protons are all equal to $\left|b_{\mathrm{HH}}\right|/(2\pi )=1$ , 7 or 15 kHz. The dipolar coupling between the ${}^{15}$ N nucleus and its closest ${}^{1}$ H neighbor is $\left|b_{\mathrm{HN}}\right|/(2\pi )=2575$ Hz, corresponding to a ${}^{1}$ H– ${}^{27}$ Al distance of 2.3 Å, typical of the distance between the protons of hydroxyl groups and the Al atoms of the first surface layer of hydrated γ -alumina (Lee et al., 2014). All protons were subject to a CSA of 6 kHz, i.e., 7.5 ppm at 18.8 T, with a null asymmetry parameter (Liang et al., 2018). We simulated the ${}^{1}$ H → ${}^{15}$ N RINEPT-CWc sequences by incorporating either $\mathrm{SR}4_{1}^{2}$ (tt) or $R12_{3}^{5}$ (tt) recoupling schemes. We used a static magnetic field of 18.8 T, for which the ${}^{1}$ H and ${}^{15}$ N Larmor frequencies were equal to 800 and 81 MHz, respectively, and MAS frequencies of $\nu_{R}=20$ or 62.5 kHz. The defocusing and refocusing periods were both equal to their optimal values τ=650 or 640 µ s at $\nu_{R}=20$ or 62.5 kHz, respectively. The rf field nutation frequency on the ${}^{1}$ H channel was equal to 200 kHz during the π/2 and π pulses that do not belong to the recoupling sequence, as well as the CW irradiation, whereas the pulses applied to $S\;={}^{15}$ N nuclei were considered ideal Dirac pulses. For the (tt) adiabatic pulses, the simulations were performed with $\nu_{1,max}/\nu_{R}$ and $\Delta \nu_{0,max}/\nu_{R}$ ratios ranging from 0.5 to 10 and from 10 to 200, respectively. All other pulses were applied on resonance. The density matrix before the first pulse was equal to $I_{1z}+I_{2z}+I_{3z}+I_{4z}$ . We normalized the transfer efficiency of the ${}^{1}$ H → ${}^{15}$ N RINEPT sequences to the maximal signal for a ${}^{1}$ H → ${}^{15}$ N through-bond RINEPT sequence made of ideal Dirac pulses in the case of a ${}^{15}$ N– ${}^{1}$ H spin system with a J -coupling constant of 150 Hz.

### 3.2 Optimal adiabatic recoupling

The transfer efficiency of RINEPT using $RN_{n}^{\nu }$ schemes built from adiabatic (tt) pulses depends on $\nu_{1,max}$ and $\Delta \nu_{0,max}$ parameters. For a similar ${}^{15}$ N ${}^{1}$ H ${}_{4}$ spin system with $\left|b_{\mathrm{HN}}\right|/(2\pi )=2.575$ and $\left|b_{\mathrm{HH}}\right|/(2\pi )=7$ kHz, spinning at $\nu_{R}=12.5$ kHz, we showed using numerical simulations that a maximal transfer efficiency was achieved provided that $\nu_{1,max}=0.07\Delta \nu_{0,max}$ and $\nu_{1,max}/\nu_{R}\geq 8$ (Nagashima et al., 2021). In practice, we used $\nu_{1,max}=11\nu_{R}=137$ kHz and $\Delta \nu_{0,max}=160\nu_{R}=2$ MHz.

**Figure 2:**
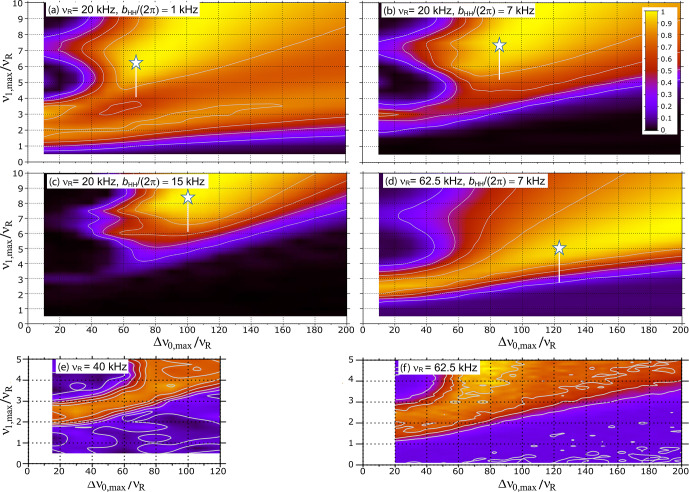
**(a–d)** Simulated transfer efficiency of ${}^{1}$ H → ${}^{15}$ N D -RINEPT- $\mathrm{SR}4_{1}^{2}(\mathrm{tt})$ sequence for a ${}^{15}$ N ${}^{1}$ H ${}_{4}$ spin system as a function of $\nu_{1,max}/\nu_{R}$ and $\Delta \nu_{0,max}/\nu_{R}$ for $\nu_{R}=20$ and 62.5 kHz and $b_{\mathrm{HH}}/(\pi )$ = **(a)** 1 kHz, **(b, d)** 7 kHz and **(c)** 15 kHz. **(e, f)** Experimental ${}^{1}$ H → ${}^{15}$ N D -RINEPT- $\mathrm{SR}4_{1}^{2}(\mathrm{tt})$ signal of L-histidine ⋅ HCl as a function of $\nu_{1,max}/\nu_{R}$ and $\Delta \nu_{0,max}/\nu_{R}$ at 18.8 T with $\nu_{R}$ = **(e)** 40 kHz or **(f)** 62.5 kHz. In panels **(a)**–**(d)** the white star indicates recoupling conditions with minimal rf field leading to maximal transfer efficiency, and the white vertical line mimics the rf field distribution within the coil.

Similar simulations were performed here for $\nu_{R}=20$ or 62.5 kHz. As seen in Fig. 2a–c, at a given MAS frequency, higher ${}^{1}$ H– ${}^{1}$ H dipolar couplings require higher rf field and broader carrier frequency sweep so that the (tt) pulses remain adiabatic in spite of the modulation of the ${}^{1}$ H– ${}^{1}$ H dipolar couplings by MAS (Nagashima et al., 2021; Kervern et al., 2007). For $\left|b_{\mathrm{HH}}\right|/(2\pi )=7$ kHz, the minimal $\nu_{1,max}/\nu_{R}$ ratio decreases for higher MAS frequencies (compare Fig. 2b and d) since the contribution of the modulation of ${}^{1}$ H– ${}^{1}$ H dipolar couplings by MAS to the first adiabaticity factor is proportional to ( $\nu_{1,max}{)}^{2}/\nu_{R}$ ; hence, $\nu_{1,max}$ values proportional to $\sqrt{\nu_{R}}$ , i.e., $\nu_{1,max}/\nu_{R}$ ratio inversely proportional to $\sqrt{\nu_{R}}$ , are sufficient to maintain the adiabaticity of the pulses (Kervern et al., 2007). Nevertheless, Fig. 2d indicates that the $\mathrm{SR}4_{1}^{2}$ (tt) recoupling requires $\nu_{1,max}\geq 313$ kHz for $\nu_{R}=62.5$ kHz, which is hardly compatible with the specifications of most 1.3 mm MAS probes. Similar transfer efficiencies were simulated for the RINEPT sequence with $R12_{3}^{5}$ (tt) recoupling scheme (not shown).

## 4 NMR experiments

### 4.1 Samples and experimental conditions

L-[U- ${}^{15}$ N]-histidine ⋅ ​​​​​​​ HCl (hereafter referred to as “histidine”) and isotopically unmodified γ -alumina were purchased from Merck, and AlPO ${}_{4}$ -14 was prepared as described previously (Antonijevic et al., 2006).

All ${}^{1}$ H → S RINEPT-CWc and PRESTO-III experiments were performed at $B_{0}=18.8$ T on Bruker BioSpin Avance NEO spectrometers equipped with double-resonance ${}^{1}$ H/X probes.

${}^{1}$ H → ${}^{15}$ N RINEPT-CWc- $\mathrm{SR}4_{1}^{2}$ (tt) experiments on histidine were performed with 1.3 and 0.7 mm MAS probes spinning at $\nu_{R}=40$ or 62.5 kHz, with defocusing and refocusing delays equal to τ = 375 and 384 µ s, respectively. The rf field of the ${}^{1}$ H π/2 and π pulses, which do not belong to the recoupling scheme, was equal to 200 kHz, that of the continuous-wave irradiation to 100 kHz, and that of the ${}^{15}$ N pulses to 62 kHz. ${}^{1}$ H decoupling with an rf field of 16 kHz was applied during the acquisition. The pulses on the ${}^{1}$ H channel were applied on resonance, whereas those on the ${}^{15}$ N channel were applied at the isotropic chemical shift of the ${}^{15}$ NH ${}^{\tau }$ signal (172 ppm). These 1D spectra resulted from averaging eight transients with a relaxation delay of 3 s. The ${}^{15}$ N isotropic chemical shifts were referenced to an aqueous saturated solution of NH ${}_{4}$ NO ${}_{3}$ using [ ${}^{15}$ N]-glycine as a secondary reference.

**Table 1:** Comparison of the performances of ${}^{1}$ H → ${}^{27}$ Al RINEPT-CWc and PRESTO transfers using various recouplings for AlO ${}_{6}$ signal of γ -alumina at $\nu_{R}=20$ kHz.

PRESTO/	Recoupling	τ	$\nu_{1}/\nu_{1,max}$	AlO ${}_{6}^{a,b}$	$\Delta \nu_{0}^{c}$	$\Delta \nu_{0}/\nu_{1}$	$\Delta \nu_{1}^{d}$	$\Delta \nu_{1}/\nu_{1}$
RINEPT		( µ s)	(kHz)		(kHz)		(kHz)	
RINEPT	$\mathrm{SR}4_{1}^{2}$ (tt)	400	160	1.00	110	0.68	> 100 ${}^{e}$	> 0.62
	$R12_{3}^{5}$ (tt)	400	160	1.00	110	0.68	> 100 ${}^{e}$	> 0.62
PRESTO	$R22_{2}^{7}$ (180 ${}_{0}$ )	400	110	0.73	30	0.27	39	0.35
RINEPT	$\mathrm{SR}4_{1}^{2}$ (270 ${}_{0}$ 90 ${}_{180}$ )	400	80	0.63	50	0.63	44	0.55
PRESTO	$R18_{2}^{5}$ (180 ${}_{0}$ )	400	90	0.61	28	0.31	27	0.30
RINEPT	$R12_{3}^{5}$ (270 ${}_{0}$ 90 ${}_{180}$ )	400	80	0.50	40	0.50	35	0.44
	$\mathrm{SR}4_{1}^{2}$ (90 ${}_{-45}$ 90 ${}_{45}$ 90 ${}_{-45}$ )	400	63	0.42	14	0.22	14	0.22
	$\mathrm{SR}4_{1}^{2}$ (180 ${}_{0}$ )	400	45	0.40	17	0.38	24	0.53
	$R12_{3}^{5}$ (180 ${}_{0}$ )	400	45	0.35	10	0.22	15	0.33
	$R12_{3}^{5}$ (90 ${}_{-45}$ 90 ${}_{45}$ 90 ${}_{-45}$ )	400	66	0.35	11	0.17	18	0.27
	$\mathrm{SC}2_{1}^{0}$	400	63	0.31	14	0.22	45	0.71
	$C6_{3}^{0}$ ( $C^{\prime }$ )	400	66	0.28	10	0.15	40	0.60

${}^{a}$ AlO ${}_{6}$ signal normalized to that with ${}^{1}$ H → ${}^{27}$ Al RINEPT-CWc- $\mathrm{SR}4_{1}^{2}$ (tt). ${}^{b}$ The relative error bars were determined from the S / N, for the AlO ${}_{6}$ signal intensity, and they are equal to ± 0.03. ${}^{c}$ FWHM (full width at half maximum) of the robustness to offset. ${}^{d}$ FWHM of the robustness to rf field. ${}^{e}$ ​​​​​​​ Only a lower bound of rf field could be determined due to probe rf specifications (Fig. 4).

**Table 2:** Comparison of the performances of ${}^{1}$ H → ${}^{27}$ Al RINEPT-CWc and PRESTO transfers with AlPO ${}_{4}$ -14 at $\nu_{R}=20$ kHz.

PRESTO/	Recoupling	τ	$\nu_{1}/\nu_{1,max}$	Intensity ${}^{a}$			$\Delta \nu_{0}$	$\Delta \nu_{0}/\nu_{1}$	$\Delta \nu_{1}$	$\Delta \nu_{1}/\nu_{1}$
RINEPT		( µ s)	(kHz)	AlO ${}_{6}^{b}$	AlO ${}_{5}^{c}$	AlO ${}_{4}^{d}$	(kHz)		(kHz)	
RINEPT	$\mathrm{SR}4_{1}^{2}$ (tt)	800	208	1.00	1.00	1.00	120	0.58	– ${}^{e}$	– ${}^{e}$
	$R12_{3}^{5}$ (tt)	800	208	0.99	0.99	0.98	120	0.58	– ${}^{e}$	– ${}^{e}$
PRESTO	$R22_{2}^{7}$ (180 ${}_{0}$ )	600	114	1.54	1.07	0.67	26	0.23	38	0.33
RINEPT	$\mathrm{SR}4_{1}^{2}$ (270 ${}_{0}$ 90 ${}_{180}$ )	800	77	0.72	0.65	0.67	45	0.58	48	0.62
PRESTO	$R18_{2}^{5}$ (180 ${}_{0}$ )	600	94	1.45	1.03	0.62	25	0.27	26	0.28
RINEPT	$R12_{3}^{5}$ (270 ${}_{0}$ 90 ${}_{180}$ )	800	77	0.58	0.50	0.48	46	0.60	36	0.47
	$\mathrm{SR}4_{1}^{2}$ (180 ${}_{0}$ )	600	43	0.64	0.45	0.36	14	0.33	23	0.53
	$\mathrm{SR}4_{1}^{2}$ (90 ${}_{-45}$ 90 ${}_{45}$ 90 ${}_{-45}$ )	800	61	0.56	0.43	0.25	16	0.26	20	0.32
	$\mathrm{SC}2_{1}^{0}$	800	68	0.54	0.41	0.24	18	0.26	52	0.73
	$R12_{3}^{5}$ (90 ${}_{-45}$ 90 ${}_{45}$ 90 ${}_{-45}$ )	600	61	0.43	0.30	0.21	8	0.13	18	0.29
	$R12_{3}^{5}$ (180 ${}_{0}$ )	600	45	0.34	0.28	0.21	8	0.18	18	0.40
	$C6_{3}^{0}$ ( $C^{\prime }$ )	600	68	0.52	0.36	0.21	10	0.15	42	0.61

${}^{a}$ Intensities of AlO ${}_{6}$ , AlO ${}_{5}$ and AlO ${}_{4}$ resonances normalized to their intensities with ${}^{1}$ H → ${}^{27}$ Al RINEPT-CWc- $\mathrm{SR}4_{1}^{2}$ (tt). The relative errors for the signal intensities are ${}^{b}$ ± 0.02, ${}^{c}$ ± 0.03 and ${}^{d}$ ± 0.01, for AlO ${}_{6}$ , AlO ${}_{5}$ and AlO ${}_{4}$ , respectively. ${}^{e}$ FWHM of the robustness to rf field was not measured for RINEPT- $\mathrm{SR}4_{1}^{2}$ (tt) or RINEPT- $R12_{3}^{5}$ (tt) (Fig. S2).

**Figure 3:**
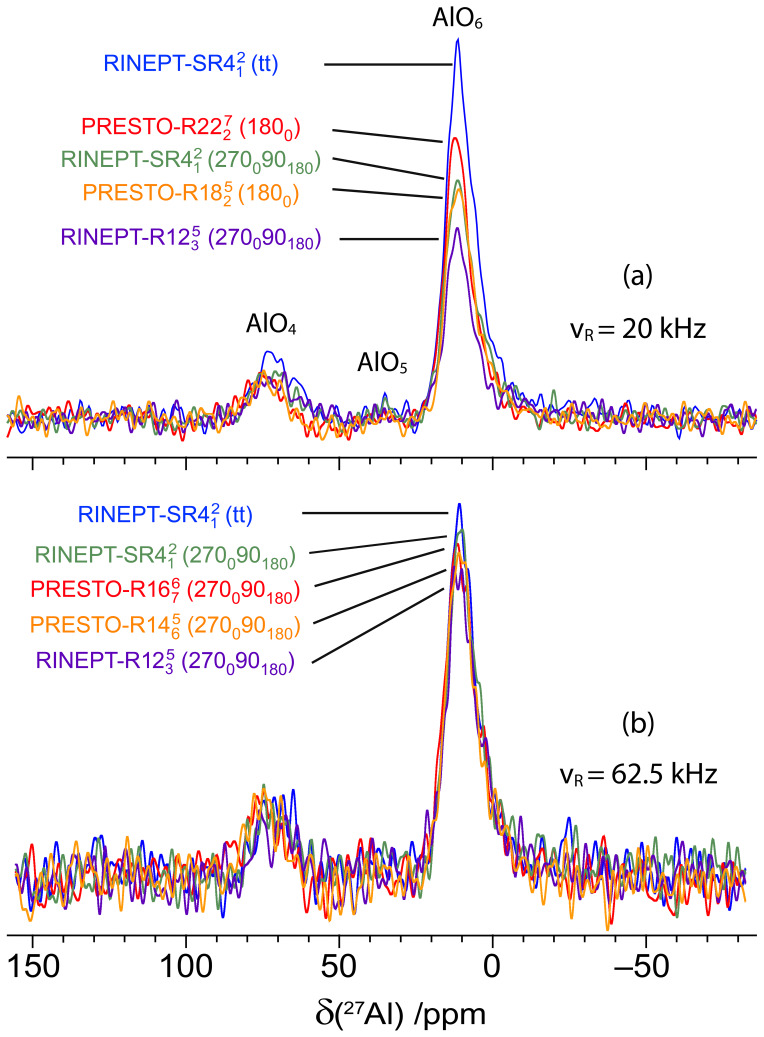
The ${}^{27}$ Al 1D spectra of γ -alumina at 18.8 T with $\nu_{R}=20$ kHz **(a)** and 62.5 kHz **(b)** acquired using ${}^{1}$ H → ${}^{27}$ Al transfers with RINEPT-CWc and $\mathrm{SR}4_{1}^{2}(\mathrm{tt})$ , $\mathrm{SR}4_{1}^{2}$ (270 ${}_{0}$ 90 ${}_{180}$ ) and $R12_{3}^{5}$ (270 ${}_{0}$ 90 ${}_{180}$ ), or PRESTO and **(a)** $R22_{2}^{7}$ (180 ${}_{0}$ ) or $R18_{2}^{5}$ (180 ${}_{0}$ ), or **(b)** $R16_{7}^{6}$ (270 ${}_{0}$ 90 ${}_{180}$ ) and $R14_{6}^{5}$ (270 ${}_{0}$ 90 ${}_{180}$ ). The τ delays and $\nu_{1}/\nu_{1,max}$ rf fields were fixed to their optimum values given in Tables 1 and 3.

**Figure 4:**
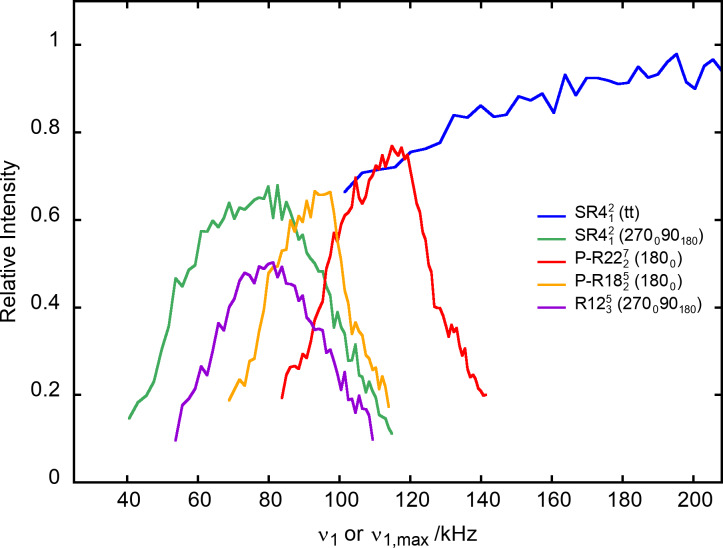
The ${}^{27}$ AlO ${}_{6}$ on-resonance signal of γ -alumina at $\nu_{R}=20$ kHz as a function of $\nu_{1}$ or $\nu_{1,max}$ for PRESTO- $R22_{2}^{7}$ (180 ${}_{0}$ ) and PRESTO- $R18_{2}^{5}$ (180 ${}_{0}$ ) as well as RINEPT-CWc- $\mathrm{SR}4_{1}^{2}$ (tt), RINEPT- $\mathrm{SR}4_{1}^{2}$ (270 ${}_{0}$ 90 ${}_{180}$ ) and RINEPT- $R12_{3}^{5}$ (270 ${}_{0}$ 90 ${}_{180}$ ). For each curve, τ was fixed to its optimum value given in Table 1.

**Figure 5:**
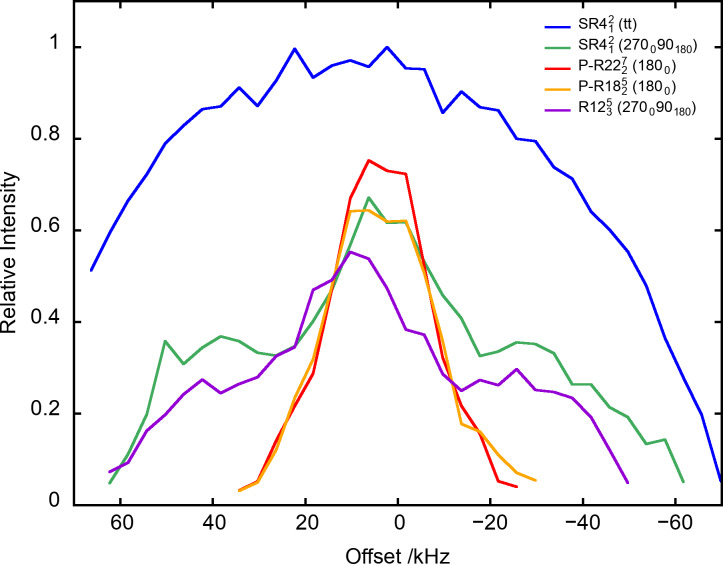
The ${}^{27}$ AlO ${}_{6}$ signal of γ -alumina at $\nu_{R}=20$ kHz as a function of offset for PRESTO- $R22_{2}^{7}$ (180 ${}_{0}$ ) and PRESTO- $R18_{2}^{5}$ (180 ${}_{0}$ ) as well as RINEPT-CWc- $\mathrm{SR}4_{1}^{2}$ (tt), RINEPT- $\mathrm{SR}4_{1}^{2}$ (270 ${}_{0}$ 90 ${}_{180}$ ) and RINEPT- $R12_{3}^{5}$ (270 ${}_{0}$ 90 ${}_{180}$ ). For each curve, τ and $\nu_{1}$ or $\nu_{1,max}$ were fixed to their optimum values given in Table 1.

**Figure 6:**
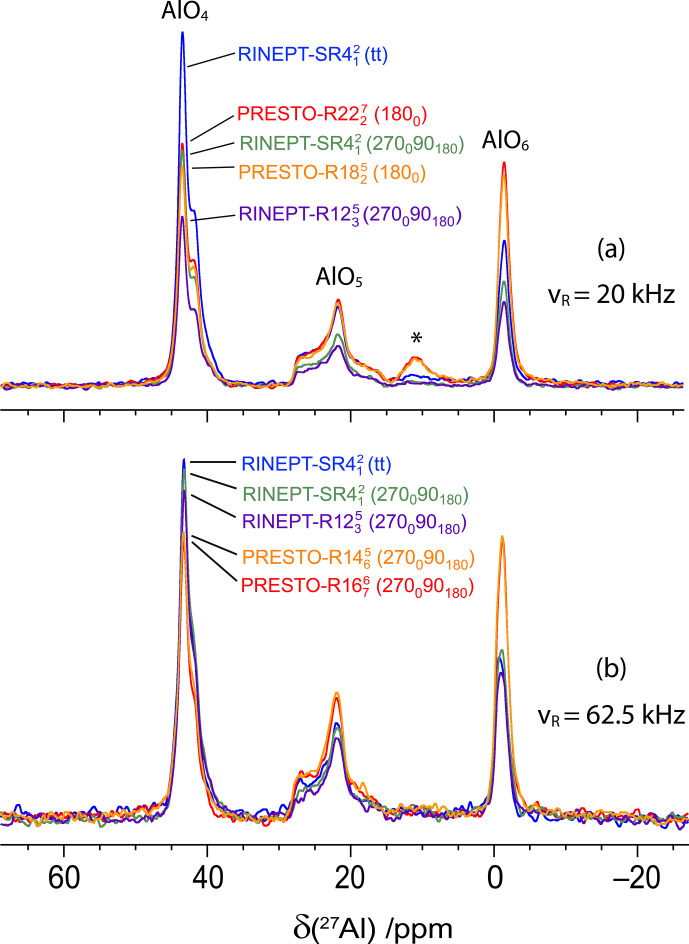
The ${}^{27}$ Al 1D spectra of AlPO ${}_{4}$ -14 at 18.8 T with $\nu_{R}=20$ kHz **(a)** and 62.5 kHz **(b)** acquired using ${}^{1}$ H → ${}^{27}$ Al transfers with RINEPT-CWc and $\mathrm{SR}4_{1}^{2}(\mathrm{tt})$ , $\mathrm{SR}4_{1}^{2}$ (270 ${}_{0}$ 90 ${}_{180}$ ) and $R12_{3}^{5}$ (270 ${}_{0}$ 90 ${}_{180}$ ), or PRESTO and **(a)** $R22_{2}^{7}$ (180 ${}_{0}$ ) and $R18_{2}^{5}$ (180 ${}_{0}$ ), or **(b)** $R16_{7}^{6}$ (270 ${}_{0}$ 90 ${}_{180}$ ) and $R14_{6}^{5}$ (270 ${}_{0}$ 90 ${}_{180}$ ). The τ delays and $\nu_{1}/\nu_{1,max}$ rf fields were fixed to their optimal values given in Tables 2 and 4. The resonance at ca. 11 ppm in panel **(a)** is due to an impurity.

**Figure 7:**
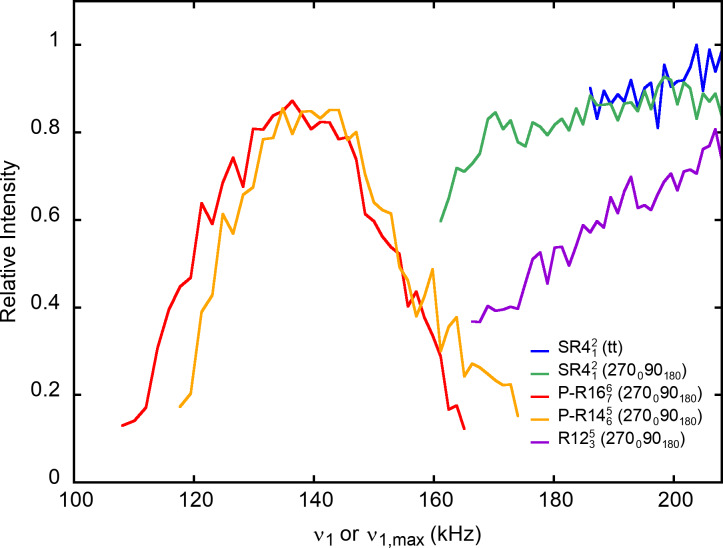
The ${}^{27}$ AlO ${}_{6}$ on-resonance signal of γ -alumina at $\nu_{R}=62.5$ kHz as a function of $\nu_{1}$ or $\nu_{1,max}$ for PRESTO- $R16_{7}^{6}$ (270 ${}_{0}$ 90 ${}_{180}$ ) and PRESTO- $R14_{6}^{5}$ (270 ${}_{0}$ 90 ${}_{180}$ ) as well as RINEPT-CWc- $\mathrm{SR}4_{1}^{2}$ (tt), RINEPT- $\mathrm{SR}4_{1}^{2}$ (270 ${}_{0}$ 90 ${}_{180}$ ) and RINEPT- $R12_{3}^{5}$ (270 ${}_{0}$ 90 ${}_{180}$ ). For each curve, τ was fixed to its optimum value given in Table 3.

**Figure 8:**
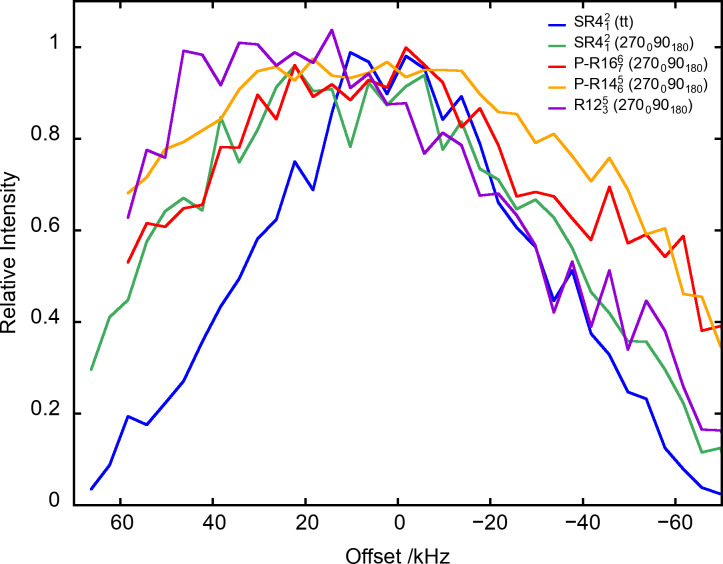
The ${}^{27}$ AlO ${}_{6}$ signal of γ -alumina at $\nu_{R}=62.5$ kHz as a function of offset for PRESTO- $R16_{7}^{6}$ (270 ${}_{0}$ 90 ${}_{180}$ ) and PRESTO- $R14_{6}^{5}$ (270 ${}_{0}$ 90 ${}_{180}$ ) as well as RINEPT-CWc- $\mathrm{SR}4_{1}^{2}$ (tt), RINEPT- $\mathrm{SR}4_{1}^{2}$ (270 ${}_{0}$ 90 ${}_{180}$ ) and RINEPT- $R12_{3}^{5}$ (270 ${}_{0}$ 90 ${}_{180}$ ). For each curve, τ and $\nu_{1}$ or $\nu_{1,max}$ were fixed to their optimum values given in Table 3.

${}^{1}$ H → ${}^{27}$ Al RINEPT-CWc and PRESTO-III experiments on γ -alumina and AlPO ${}_{4}$ -14 were performed with a 1.3 mm MAS probe spinning at $\nu_{R}=20$ (to test the $RN_{n}^{\nu }$ schemes with large rf field requirement) or 62.5 kHz. The tested recoupling schemes are listed in Tables 1 and 2 for $\nu_{R}=20$ kHz and Tables 3 and 4 for $\nu_{R}=62.5$ kHz. The rf field of the ${}^{1}$ H π/2 and π pulses, which do not belong to the recoupling scheme, was equal to 208 kHz, that of the continuous-wave irradiation to 147 kHz, and the ${}^{27}$ Al CT-selective one for π/2 and π pulses to 10 kHz. The defocusing and refocusing delays τ are given in Tables 1 to 4. The pulses on the ${}^{1}$ H channel were applied on resonance, whereas those on ${}^{27}$ Al channel were applied (i) on resonance with AlO ${}_{6}$ signal of γ -alumina in Figs. 4 and 7, Tables 1 and 3, and in Figs. 5 and 8 when the offset is null; (ii) on resonance with AlO ${}_{4}$ signal of AlPO ${}_{4}$ -14 in Figs. S2 and S4, Tables 2 and 4 as well as in Figs. S3 and S5 when the offset is null; and (iii) in the middle of the AlO ${}_{4}$ and AlO ${}_{6}$ peaks for the 1D spectra shown in Figs. 3 and 6. These differences in offset explain some changes in the relative efficiencies of the recoupling between the figures. These 1D spectra resulted from averaging 64 transients with a relaxation delay of 1 s. The ${}^{27}$ Al isotropic chemical shifts were referenced at 0 ppm to 1 mol L ${}^{-1}$ [Al(H ${}_{2}$ O) ${}_{6}$ ] ${}^{3+}$ solution.

**Table 3:** Comparison of the performances of ${}^{1}$ H → ${}^{27}$ Al RINEPT-CWc and PRESTO transfers using various recouplings for the AlO ${}_{6}$ signal of γ -alumina at $\nu_{R}=62.5$ kHz.

PRESTO/	Recoupling	τ	$\nu_{1}/\nu_{1,max}$	AlO ${}_{6}^{a,b}$	$\Delta \nu_{0}$	$\Delta \nu_{0}/\nu_{1}$	$\Delta \nu_{1}$	$\Delta \nu_{1}/\nu_{1}$
RINEPT		( µ s)	(kHz)		(kHz)		(kHz)	
RINEPT	$\mathrm{SR}4_{1}^{2}$ (tt)	256	208	1.00	74	0.36	– ${}^{c}$	– ${}^{c}$
	$R12_{3}^{5}$ (tt)	256	208	1.00	74	0.36	– ${}^{c}$	– ${}^{c}$
	$\mathrm{SR}4_{1}^{2}$ (270 ${}_{0}$ 90 ${}_{180}$ )	320	208	0.92	96	0.46	– ${}^{c}$	– ${}^{c}$
PRESTO	$R16_{7}^{6}$ (270 ${}_{0}$ 90 ${}_{180}$ )	448	137	0.91	90	0.66	42	0.31
	$R14_{6}^{5}$ (270 ${}_{0}$ 90 ${}_{180}$ )	384	146	0.86	100	0.68	38	0.26
RINEPT	$R12_{3}^{5}$ (270 ${}_{0}$ 90 ${}_{180}$ )	320	208	0.82	86	0.41	– ${}^{c}$	– ${}^{c}$
	$\mathrm{SR}4_{1}^{2}$ (180 ${}_{0}$ )	320	125	0.75	52	0.42	88	0.70
	$R12_{3}^{5}$ (180 ${}_{0}$ )	288	125	0.74	16	0.13	85	0.68
PRESTO	$R22_{4}^{3}$ (180 ${}_{0}$ )	256	157	0.67	68	0.43	20	0.13
	$R16_{3}^{2}$ (180 ${}_{0}$ )	384	155	0.51	48	0.31	40	0.26
RINEPT	$\mathrm{SC}2_{1}^{0}$	256	186	0.34	50	0.27	84	0.45
	$C6_{3}^{0}$ ( $C^{\prime }$ )	256	186	0.34	43	0.23	76	0.41
	$\mathrm{SR}4_{1}^{2}$ (90 ${}_{-45}$ 90 ${}_{45}$ 90 ${}_{-45}$ )	256	186	0.32	47	0.25	70	0.38
	$R12_{3}^{5}$ (90 ${}_{-45}$ 90 ${}_{45}$ 90 ${}_{-45}$ )	256	186	0.32	40	0.22	70	0.38

${}^{a}$ AlO ${}_{6}$ signal normalized to that with ${}^{1}$ H → ${}^{27}$ Al RINEPT-CWc- $\mathrm{SR}4_{1}^{2}$ (tt). ${}^{b}$ The relative error on AlO ${}_{6}$ signal intensity is ± 0.08. ${}^{c}$ FWHM of the robustness to rf field was not measured for RINEPT- $\mathrm{SR}4_{1}^{2}$ (tt) or RINEPT- $R12_{3}^{5}$ (tt) (Fig. 7).

**Table 4:** Comparison of the performances of ${}^{1}$ H → ${}^{27}$ Al RINEPT-CWc and PRESTO transfers using various recouplings for AlPO ${}_{4}$ -14 at $\nu_{R}=62.5$ kHz.

PRESTO/	Recoupling	τ	$\nu_{1}/\nu_{1,max}$	Intensity ${}^{a}$			$\Delta \nu_{0}$	$\Delta \nu_{0}/\nu_{1}$	$\Delta \nu_{1}$	$\Delta \nu_{1}/\nu_{1}$
RINEPT		( µ s)	(kHz)	AlO ${}_{6}^{b}$	AlO ${}_{5}^{c}$	AlO ${}_{4}^{d}$	(kHz)		(kHz)	
RINEPT	$\mathrm{SR}4_{1}^{2}$ (tt)	480	208	1.00	1.00	1.00	48	0.23	– ${}^{e}$	– ${}^{e}$
	$R12_{3}^{5}$ (tt)	480	208	1.07	1.00	1.06	44	0.21	– ${}^{e}$	– ${}^{e}$
	$\mathrm{SR}4_{1}^{2}$ (270 ${}_{0}$ 90 ${}_{180}$ )	480	208	1.05	0.95	0.97	85	0.41	90	0.43
	$R12_{3}^{5}$ (270 ${}_{0}$ 90 ${}_{180}$ )	480	208	0.91	0.84	0.91	80	0.38	68	0.33
PRESTO	$R16_{7}^{6}$ (270 ${}_{0}$ 90 ${}_{180}$ )	672	146	1.71	1.21	0.76	80	0.55	50	0.34
	$R14_{6}^{5}$ (270 ${}_{0}$ 90 ${}_{180}$ )	576	146	1.72	1.27	0.76	86	0.59	45	0.31
RINEPT	$\mathrm{SR}4_{1}^{2}$ (180 ${}_{0}$ )	480	129	0.84	0.79	0.75	48	0.37	64	0.49
	$R12_{3}^{5}$ (180 ${}_{0}$ )	480	136	0.72	0.67	0.74	18	0.13	54	0.40
PRESTO	$R22_{4}^{3}$ (180 ${}_{0}$ )	512	157	1.47	1.18	0.69	60	0.38	20	0.33
	$R16_{3}^{2}$ (180 ${}_{0}$ )	480	147	1.17	0.83	0.52	64	0.44	20	0.31
RINEPT	$R12_{3}^{5}$ (90 ${}_{-45}$ 90 ${}_{45}$ 90 ${}_{-45}$ )	256	190	0.48	0.27	0.14	32	0.17	75	0.39
	$C6_{3}^{0}$ ( $C^{\prime }$ )	256	193	0.47	0.28	0.14	28	0.15	78	0.40
	$\mathrm{SR}4_{1}^{2}$ (90 ${}_{-45}$ 90 ${}_{45}$ 90 ${}_{-45}$ )	256	196	0.48	0.14	0.14	36	0.18	77	0.39
	$\mathrm{SC}2_{1}^{0}$	256	188	0.53	0.25	0.14	44	0,23	80	0.43

${}^{a}$ Intensities of AlO ${}_{6}$ , AlO ${}_{5}$ and AlO ${}_{4}$ resonances normalized to their intensities with ${}^{1}$ H → ${}^{27}$ Al RINEPT-CWc- $\mathrm{SR}4_{1}^{2}$ (tt). The relative errors for the signal intensities are ${}^{b}$ ± 0.04, ${}^{c}$ ± 0.06 and ${}^{d}$ ± 0.02, for AlO ${}_{6}$ , AlO ${}_{5}$ and AlO ${}_{4}$ , respectively. ${}^{e}$ FWHM of the robustness to rf field was not measured for RINEPT- $\mathrm{SR}4_{1}^{2}$ (tt) or RINEPT- $R12_{3}^{5}$ (tt) (Fig. S4).

We also measured the decay of the transverse proton magnetization of AlPO ${}_{4}$ -14 during a spin echo sequence, in which the refocusing π pulse was identical to that used in the defocusing part of the RINEPT-CWc sequences (Fig. 1b). This decay was measured at $\nu_{R}=20$ and 62.5 kHz either without any recoupling or by applying a $\mathrm{SR}4_{1}^{2}$ recoupling built from (180 ${}_{0}$ ), (270 ${}_{0}$ 90 ${}_{180}$ ) and (tt) pulses during the delays of the spin echo sequence. The rf fields during the recoupling two blocks were equal to their optimal values given in Tables 2 and 4.

We also acquired several 2D ${}^{1}$ H → ${}^{27}$ Al D -HETCOR spectra of AlPO ${}_{4}$ -14 using RINEPT-CWc- $\mathrm{SR}4_{1}^{2}$ with (180 ${}_{0}$ ), (270 ${}_{0}$ 90 ${}_{180}$ ) and (tt) pulses as well as PRESTO- $R16_{7}^{6}$ (270 ${}_{0}$ 90 ${}_{180}$ ). These 2D spectra were acquired using a non-uniform sampling (NUS) with an exponentially biased sampling retaining 25 % of the points with respect to uniform sampling. The 2D spectra resulted from eight transients for each of the 500 $t_{1}$ increments with a recycle delay of 1 s, i.e., an acquisition time of 72 min.

### 4.2 Optimal adiabatic recoupling

Figure 2e and f show the efficiency of the ${}^{1}$ H → ${}^{15}$ N RINEPT- $\mathrm{SR}4_{1}^{2}$ (tt) transfer for histidine as a function of the $\nu_{1,max}/\nu_{R}$ and $\Delta \nu_{0,max}/\nu_{R}$ ratios for $\nu_{R}$ = 40 or 62.5 kHz, respectively. These experimental data indicate that at higher MAS frequencies, an efficient adiabatic recoupling can be achieved for lower $\nu_{1,max}/\nu_{R}$ and $\Delta \nu_{0,max}/\nu_{R}$ ratios. This result agrees with the numerical simulations of Fig. 2b and d.

### 4.3 PRESTO and RINEPT performances for $\nu_{R}=20$ kHz

#### 4.3.1 γ -alumina

The 1D spectra of γ -alumina acquired using ${}^{1}$ H → ${}^{27}$ Al RINEPT and PRESTO sequences, shown in Fig. 3, exhibit two resonances at 70 and 10 ppm, assigned to tetra- (AlO ${}_{4}$ ) and hexa-coordinated (AlO ${}_{6}$ ) resonances, respectively (Morris and Ellis, 1989). The signal of penta-coordinated (AlO ${}_{5}$ ) sites, which are mainly located in the first surface layer, is barely detected because of the lack of sensitivity of conventional solid-state NMR spectroscopy (Lee et al., 2014). The most intense peak, AlO ${}_{6}$ , was used to compare the transfer efficiencies of RINEPT and PRESTO sequences with different recoupling schemes. Table 1 lists the measured performances of ${}^{1}$ H → ${}^{27}$ Al RINEPT-CWc and PRESTO transfers using various recoupling for γ -alumina at $\nu_{R}=20$ kHz. We notably compared the PRESTO sequences using $R22_{2}^{7}$ (180 ${}_{0}$ ) and $R18_{2}^{5}$ (180 ${}_{0}$ ) recoupling with the RINEPT-CWc scheme using $\mathrm{SR}4_{1}^{2}$ and $R12_{3}^{5}$ with single (180 ${}_{0}$ ), composite (270 ${}_{0}$ 90 ${}_{180}$ ), and (90 ${}_{-45}$ 90 ${}_{45}$ 90 ${}_{-45}$ ) or (tt) adiabatic pulses. A low transfer efficiency was obtained for RINEPT-CWc- $\mathrm{SR}4_{1}^{2}$ (90 ${}_{0}$ 240 ${}_{90}$ 90 ${}_{0}$ ) because of its low scaling factor, κ = 0.131; hence, its performances are not reported in Table 1. We also tested the recoupling schemes based on the symmetry $\mathrm{SC}2_{1}^{0}$ , corresponding to the ${\left[C2_{1}^{0}\right]}_{0}{\left[C2_{1}^{0}\right]}_{120}{\left[C2_{1}^{0}\right]}_{240}$ sequence with a basic element C = (90 ${}_{45}$ 90 ${}_{135}$ 90 ${}_{45}$ 90 ${}_{225}$ 90 ${}_{315}$ 90 ${}_{225}$ ) or $C6_{3}^{0}$ built from $C^{\prime }$ = (90 ${}_{30}$ 90 ${}_{120}$ 90 ${}_{30}$ 90 ${}_{240}$ 90 ${}_{330}$ 90 ${}_{240}$ ). These basic elements, which derive from (90 ${}_{-45}$ 90 ${}_{45}$ 90 ${}_{-45}$ ), have recently been proposed (Perras et al., 2019). As seen in Table 1 and Fig. 3a, the sequences yielding the highest transfer efficiencies are by decreasing order RINEPT-CWc with $\mathrm{SR}4_{1}^{2}$ (tt) or $R12_{3}^{5}$ (tt) > PRESTO- $R22_{2}^{7}$ (180 ${}_{0}$ ) > RINEPT-CWc- $\mathrm{SR}4_{1}^{2}$ (270 ${}_{0}$ 90 ${}_{180}$ ) ≈ PRESTO- $R18_{2}^{5}$ (180 ${}_{0}$ ) > RINEPT-CWc- $R12_{3}^{5}$ (270 ${}_{0}$ 90 ${}_{180}$ ). Figures 4 and 5 display the signal intensity of these sequences as a function of the rf field amplitude and offset, respectively.

The highest transfer efficiencies are obtained with the RINEPT-CWc sequence incorporating a (tt) adiabatic pulse. This recoupling also leads to the highest robustness to offset and rf inhomogeneity, and $\mathrm{SR}4_{1}^{2}$ (tt) and $R12_{3}^{5}$ (tt) yield identical transfer efficiency and robustness. Hence, the three-step multiple-quantum super-cycle of the $\mathrm{SR}4_{1}^{2}$ symmetry does not improve the robustness in the case of a (tt) basic element. However, these recoupling schemes require maximum rf fields of $\nu_{1,max}\geq 8\nu_{R}=$ 160 kHz, which may exceed the rf power specifications of most 3.2 mm MAS probes.

The PRESTO sequences using $R22_{2}^{7}$ (180 ${}_{0}$ ) and $R18_{2}^{5}$ (180 ${}_{0}$ ) recoupling also result in good transfer efficiencies but lower than RINEPT-CWc- $\mathrm{SR}4_{1}^{2}$ (tt). However, they use rf fields of $\nu_{1}/\nu_{R}=$ 5.5 and 4.5, which are compatible with the specifications of 3.2 mm MAS probes. The higher transfer efficiency of $R22_{2}^{7}$ (180 ${}_{0}$ ) with respect to $R18_{2}^{5}$ (180 ${}_{0}$ ) stems from its weaker second-order cross terms between ${}^{1}$ H– ${}^{1}$ H interactions (Table S1).

The efficiency of the RINEPT-CWc- $\mathrm{SR}4_{1}^{2}$ (270 ${}_{0}$ 90 ${}_{180}$ ) sequence, with $\nu_{1}=4\nu_{R}$ , is comparable to that of PRESTO- $R18_{2}^{5}$ (180 ${}_{0}$ ) but with a higher robustness to offset and rf inhomogeneity. We can notice that amplitude modulated recoupling schemes, for which the phase shifts are equal to 180 ${}^{\circ }$ , such as $\mathrm{SR}4_{1}^{2}$ (270 ${}_{0}$ 90 ${}_{180}$ ) and $\mathrm{SR}4_{1}^{2}$ (180 ${}_{0}$ ), exhibit a high robustness to rf field maladjustments (Fig. 5) (Carravetta et al., 2000). The use of (270 ${}_{0}$ 90 ${}_{180}$ ) composite pulses with $\mathrm{SR}4_{1}^{2}$ symmetry, instead of single π pulses, improves its transfer efficiency as well as its robustness to offset and rf field inhomogeneity.

In summary, for $\nu_{R}=20$ kHz in γ -alumina, the RINEPT-CWc- $\mathrm{SR}4_{1}^{2}$ (270 ${}_{0}$ 90 ${}_{180}$ ) sequence achieves efficient and robust transfers of magnetization from protons to ${}^{27}$ Al nuclei using a moderate rf field of $\nu_{1}=4\nu_{R}$ . For ${}^{1}$ H spectra with a width smaller than 20 kHz and MAS probes with a good rf homogeneity, PRESTO- $R22_{2}^{7}$ (180 ${}_{0}$ ) can result in slightly higher transfer efficiencies.

#### 4.3.2 AlPO ${}_{4}$ -14

Figure 6a shows the ${}^{1}$ H → ${}^{27}$ Al RINEPT and PRESTO 1D spectra of AlPO ${}_{4}$ -14 recorded with $\nu_{R}=20$ kHz. They exhibit three ${}^{27}$ Al resonances at 43, 21 and - 2 ppm assigned to AlO ${}_{4}$ , AlO ${}_{5}$ and AlO ${}_{6}$ sites, respectively (Ashbrook et al., 2008) The AlO ${}_{5}$ and AlO ${}_{6}$ sites are directly bonded to OH groups. The ${}^{1}$ H MAS spectrum is shown in Fig. S1. According to the literature, the ${}^{27}$ AlO ${}_{4}$ signal subsumes the resonances of two AlO ${}_{4}$ sites with quadrupolar coupling constants $C_{Q}$ = 1.7 and 4.1 MHz, whereas those of AlO ${}_{5}$ and AlO ${}_{6}$ sites are equal to 5.6 and 2.6 MHz, respectively (Fernandez et al., 1996; Antonijevic et al., 2006). The ${}^{1}$ H– ${}^{1}$ H dipolar couplings within the isopropylamine template molecule are larger than in γ -alumina. We used the most intense peak, AlO ${}_{4}$ , to compare the ${}^{1}$ H → ${}^{27}$ Al transfer efficiencies of RINEPT-CWc and PRESTO sequences with different recoupling schemes, and the results are given in Table 2. The six sequences yielding the highest transfer efficiencies are the same as for γ -alumina and their relative efficiencies are comparable for the AlO ${}_{4}$ peak of AlPO ${}_{4}$ -14 and the AlO ${}_{6}$ signal of γ -alumina.

Nevertheless, the rf requirement of the $\mathrm{SR}4_{1}^{2}$ (tt) and $R12_{3}^{5}$ (tt) schemes is higher for AlPO ${}_{4}$ -14 than for γ -alumina because of the larger ${}^{1}$ H– ${}^{1}$ H dipolar couplings, in agreement with the numerical simulations of Fig. 2a–c. This rf requirement prevents the use of these adiabatic recoupling schemes at $\nu_{R}=20$ kHz with most 3.2 mm MAS probes. That of the other sequences and their robustness to offset and rf field homogeneity are similar for both samples (Table 2 and Figs. S2 and S3).

In the case of AlPO ${}_{4}$ -14, PRESTO yields a higher efficiency than RINEPT for AlO ${}_{5}$ and AlO ${}_{6}$ , contrary to the AlO ${}_{4}$ resonance, since (i) these Al sites are directly bonded to OH groups and (ii) $R22_{2}^{7}$ (180 ${}_{0}$ ) and $R18_{2}^{5}$ (180 ${}_{0}$ ) schemes are subject to dipolar truncation (Sect. 2.1.1), which prevents to transfer the ${}^{1}$ H magnetization of these OH groups to ${}^{27}$ AlO ${}_{4}$ nuclei.

Hence, at $\nu_{R}=20$ kHz, for both AlPO ${}_{4}$ -14 and γ -alumina, the RINEPT-CWc- $\mathrm{SR}4_{1}^{2}$ (270 ${}_{0}$ 90 ${}_{180}$ ) and PRESTO- $R22_{2}^{7}$ (180 ${}_{0}$ ) sequences are the best choices to transfer the ${}^{1}$ H magnetization to ${}^{27}$ Al nuclei.

### 4.4 PRESTO and RINEPT performances for $\nu_{R}=62.5$ kHz

Similar comparisons of the performances of the various RINEPT-CWc and PRESTO sequences were performed for γ -alumina and AlPO ${}_{4}$ -14 at $\nu_{R}=62.5$ kHz.

#### 4.4.1 γ -alumina

The corresponding data for γ -alumina are given in Table 3. The sequences yielding the highest transfer efficiencies are by decreasing order: RINEPT-CWc with $\mathrm{SR}4_{1}^{2}$ (tt) or $R12_{3}^{5}$ (tt) > RINEPT-CWc- $\mathrm{SR}4_{1}^{2}$ (270 ${}_{0}$ 90 ${}_{180}$ ) ≈ PRESTO- $R16_{7}^{6}$ (270 ${}_{0}$ 90 ${}_{180}$ ) > PRESTO- $R14_{6}^{5}$ (270 ${}_{0}$ 90 ${}_{180}$ ) > RINEPT-CWc- $R12_{3}^{5}$ (270 ${}_{0}$ 90 ${}_{180}$ ).

The nominal rf requirements of the RINEPT sequences using adiabatic or (270 ${}_{0}$ 90 ${}_{180}$ ) composite π pulses correspond to $\nu_{1,max}\approx 5\nu_{R}$ (313 kHz: Fig. 2d) or 4 $\nu_{R}$ (250 kHz), which exceed the specifications of our 1.3 mm MAS probe, and the sequences were tested only up to $\nu_{1,max}=208$ kHz (Fig. 7). This suboptimal rf field may limit the transfer efficiencies of these sequences.

The PRESTO- $R16_{7}^{6}$ (270 ${}_{0}$ 90 ${}_{180}$ ) and PRESTO- $R14_{6}^{5}$ (270 ${}_{0}$ 90 ${}_{180}$ ) sequences yield transfer efficiencies comparable to those of RINEPT-CWc- $\mathrm{SR}4_{1}^{2}$ (270 ${}_{0}$ 90 ${}_{180}$ ) but with a significantly lower rf field, $\nu_{1}\approx 137$ kHz $\approx 2.3\nu_{R}$ . Furthermore, the robustness to offset of these PRESTO sequences is comparable to that of RINEPT-CWc- $\mathrm{SR}4_{1}^{2}$ (270 ${}_{0}$ 90 ${}_{180}$ ) (Fig. 8). PRESTO- $R22_{4}^{3}$ (180 ${}_{0}$ ) and PRESTO- $R16_{3}^{2}$ (180 ${}_{0}$ ) sequences with a small phase shift of 2ϕ≤52 ${}^{\circ }$ are less efficient, because they are sensitive to rf inhomogeneity.

#### 4.4.2 AlPO ${}_{4}$ -14

In the case of AlPO ${}_{4}$ -14, the relative transfer efficiencies for ${}^{27}$ AlO ${}_{4}$ species follow a similar order as for γ -alumina, except that the transfer efficiencies of PRESTO- $R16_{7}^{6}$ (270 ${}_{0}$ 90 ${}_{180}$ ) and PRESTO- $R14_{6}^{5}$ (270 ${}_{0}$ 90 ${}_{180}$ ) are significantly lower than that of RINEPT-CWc- $\mathrm{SR}4_{1}^{2}$ (270 ${}_{0}$ 90 ${}_{180}$ ) (Table 4). This decreased efficiency of the PRESTO schemes for AlO ${}_{4}$ stems notably from the dipolar truncation, which prevents the transfer of magnetization from the OH groups bonded to AlO ${}_{5}$ and AlO ${}_{6}$ sites to AlO ${}_{4}$ , since these ${}^{27}$ AlO ${}_{4}$ nuclei are significantly more distant from protons (see Table S5). Furthermore, the amplitude-modulated $\mathrm{SR}4_{1}^{2}$ (270 ${}_{0}$ 90 ${}_{180}$ ) recoupling benefits from a higher robustness to rf field inhomogeneity than the PRESTO schemes (Fig. S4). Conversely, the robustness to offset of these three sequences are comparable (Fig. S5), whereas the rf requirements of $R16_{7}^{6}$ (270 ${}_{0}$ 90 ${}_{180}$ ) and $R14_{6}^{5}$ (270 ${}_{0}$ 90 ${}_{180}$ ) are much lower than that of $\mathrm{SR}4_{1}^{2}$ (270 ${}_{0}$ 90 ${}_{180}$ ).

In summary, at $\nu_{R}=62.5$ kHz, for both γ -alumina and isopropylamine-templated AlPO ${}_{4}$ -14, PRESTO- $R16_{7}^{6}$ (270 ${}_{0}$ 90 ${}_{180}$ ) and RINEPT-CWc- $\mathrm{SR}4_{1}^{2}$ (270 ${}_{0}$ 90 ${}_{180}$ ) are the best methods to transfer the polarization of protons to quadrupolar nuclei. However, the first sequence requires a much lower rf field than the second does.

### 4.5 Decay of transverse ${}^{1}$ H magnetization during recoupling

We also measured the decay of the ${}^{1}$ H transverse magnetization during a spin echo experiment, in which the refocusing π pulse was the composite one employed in the defocusing part of the RINEPT-CW sequence shown in Fig. 1b. We performed these experiments on AlPO ${}_{4}$ -14 since the ${}^{1}$ H– ${}^{1}$ H dipolar interactions are larger in this sample than in γ -alumina. This decay was measured either in the absence of any recoupling or under a $\mathrm{SR}4_{1}^{2}$ recoupling built from (180 ${}_{0}$ ), (270 ${}_{0}$ 90 ${}_{180}$ ) or (tt) inversion element. The three ${}^{1}$ H signals featured a mono-exponential decay with a time constant $T_{2}^{\prime }$ reported in Table 5.

**Table 5:** The ${}^{1}$ H $T_{2}^{\prime }$ values of AlPO ${}_{4}$ -14 without recoupling or with $\mathrm{SR}4_{1}^{2}$ recoupling built from (180 ${}_{0}$ ), (270 ${}_{0}$ 90 ${}_{180}$ ) or (tt) inversion elements. The estimated error bars are equal to 7 %.

$\nu_{R}$ (kHz)	20			62.5		
$T_{2}^{\prime }$ (ms)	NH ${}_{3}^{+}$	CH	CH ${}_{3}+$ OH	NH ${}_{3}^{+}$	CH	CH ${}_{3}+$ OH
No recoupling	1.6	1.6	1.4	4.0	4.2	4.4
(180 ${}_{0}$ )	0.6	0.4	0.6	0.9	0.5	0.7
(270 ${}_{0}$ 90 ${}_{180}$ )	0.8	0.6	0.9	0.5	0.3	0.4
(tt)	52	1000	170	2.2	2.7	2.1

At $\nu_{R}=20$ kHz, the $T_{2}^{\prime }$ constants are significantly shorter under $\mathrm{SR}4_{1}^{2}$ (180 ${}_{0}$ ) and $\mathrm{SR}4_{1}^{2}$ (270 ${}_{0}$ 90 ${}_{180}$ ) than without recoupling. This faster decay can stem from the reintroduction of ${}^{1}$ H– ${}^{1}$ H dipolar interactions in the second- and higher-order terms of the AH by the recoupling as well as the effect of pulse transients (Wittmann et al., 2016). Conversely, the $T_{2}^{\prime }$ constants under $\mathrm{SR}4_{1}^{2}$ (tt) are much longer than without recoupling, showing that the adiabatic pulses using large rf field efficiently decouple the ${}^{1}$ H– ${}^{1}$ H dipolar interactions, whereas the continuous variation of the phase and amplitude during these pulses minimizes the transients.

At $\nu_{R}=62.5$ kHz, the $T_{2}^{\prime }$ constants without recoupling are lengthened with respect to those at $\nu_{R}=20$ kHz since faster MAS better averages the ${}^{1}$ H– ${}^{1}$ H dipolar interactions (Mao et al., 2009). Conversely, the $T_{2}^{\prime }$ constants under $\mathrm{SR}4_{1}^{2}$ (270 ${}_{0}$ 90 ${}_{180}$ ) recoupling are shorter at $\nu_{R}=62.5$ than at 20 kHz. This counterintuitive reduction may stem from the shorter pulse lengths at $\nu_{R}=62.5$ kHz, which result in a larger number of transients. Indeed, the recoupling time, τ , only depends on the sample; hence, the number of transients is proportional to $\nu_{R}$ , because the recoupling sequences are rotor synchronized. Moreover, it also increases with the use of composite pulses and as a result there are 6.25 times more transients at $\nu_{R}=62.5$ kHz and (270 ${}_{0}$ ,90 ${}_{180}$ ) pulses than at 20 kHz MAS and (180 ${}_{0}$ ) pulses. Additionally, the rf power increases with the spinning speed and the use of composite pulses, and then also the amplitude of the transients. For the same reason, the $T_{2}^{\prime }$ constants under $\mathrm{SR}4_{1}^{2}$ (180 ${}_{0}$ ) are only slightly longer at high MAS frequency. The $T_{2}^{\prime }$ constants under $\mathrm{SR}4_{1}^{2}$ (tt) recoupling are much shorter at $\nu_{R}=62.5$ than at 20 kHz, because the adiabaticity criterion is not fulfilled at $\nu_{R}=62.5$ kHz; hence, the elimination of ${}^{1}$ H– ${}^{1}$ H dipolar interactions is less effective (Figs. 2f and S4).

### 4.6 2D ${}^{1}$ H– ${}^{27}$ Al D -HETCOR of AlPO ${}_{4}$ -14

**Figure 9:**
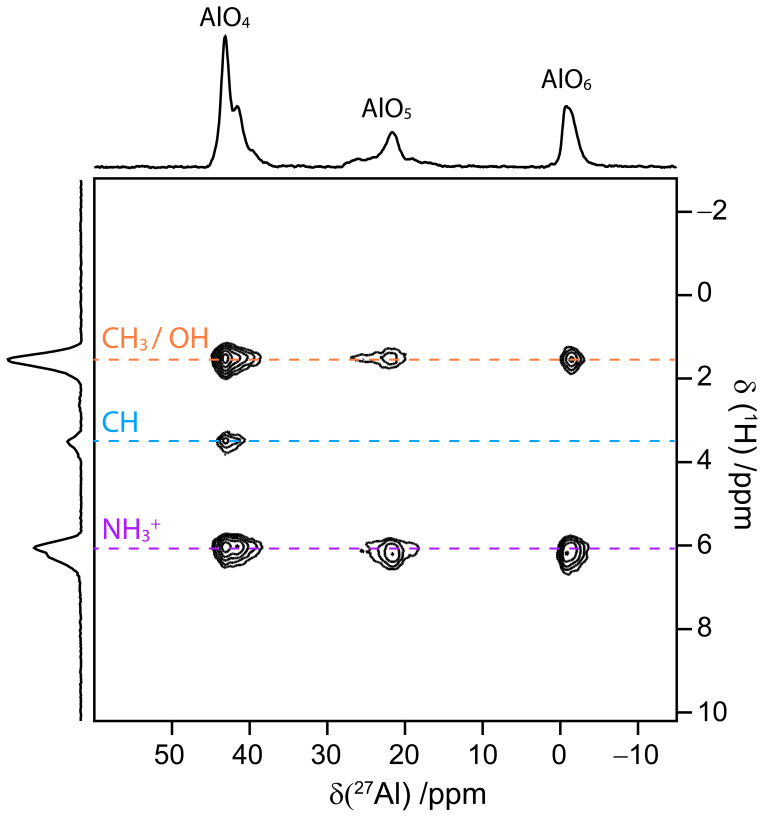
The ${}^{1}$ H– ${}^{27}$ Al D -HETCOR 2D spectrum of AlPO ${}_{4}$ -14, along with its skyline projections, at $B_{0}$ = 18.8 T and $\nu_{R}=62.5$ kHz acquired in only 72 min with only ca. 2.5 µ L of active volume with NUS 25 % using RINEPT-CWc- $\mathrm{SR}4_{1}^{2}$ (270 ${}_{0}$ 90 ${}_{180}$ ) transfer.

Figure 9 demonstrates the possibility to acquire 2D ${}^{1}$ H– ${}^{27}$ Al D -HETCOR spectra using RINEPT-CWc- $\mathrm{SR}4_{1}^{2}$ (270 ${}_{0}$ ,90 ${}_{180}$ ) transfer at $\nu_{R}=62.5$ kHz. This spectrum was recorded using a NUS scheme retaining 25 % of the $t_{1}$ points, which would be acquired using uniform sampling. In this spectrum, the CH proton only correlates with the AlO ${}_{4}$ site since it is too distant from AlO ${}_{5}$ and AlO ${}_{6}$ sites (see Table S5). The other two ${}^{1}$ H signals correlate with the three Al environments. Similar 2D spectra (not shown) were acquired using RINEPT-CWc transfer based on $\mathrm{SR}4_{1}^{2}$ (180 ${}_{0}$ ) and $\mathrm{SR}4_{1}^{2}$ (tt) recoupling as well as PRESTO- $R16_{7}^{6}$ (270 ${}_{0}$ 90 ${}_{180}$ ). Their skyline projections are shown in Figs. S6 and S7.

## 5 Conclusions

In this work, we have introduced novel symmetry-based heteronuclear dipolar recoupling schemes, which can be incorporated into the RINEPT and PRESTO sequences to transfer the magnetization from protons to half-integer quadrupolar nuclei at $\nu_{R}=20$ or 62.5 kHz. These new recouplings have been compared to the existing ones. We have shown that the RINEPT-CWc- $\mathrm{SR}4_{1}^{2}$ (tt) sequence with adiabatic pulses, which produces efficient and robust transfers at $\nu_{R}\approx 10$ –15 kHz (Nagashima et al., 2020), requires rf fields incompatible with the specifications of most MAS probes for $\nu_{R}\geq 20$ kHz. Conversely, the introduced RINEPT-CWc- $\mathrm{SR}4_{1}^{2}$ (270 ${}_{0}$ 90 ${}_{180}$ ) and PRESTO- $R22_{2}^{7}$ (180 ${}_{0}$ ) techniques with rf fields of ca. 4 $\nu_{R}$ and 5.5 $\nu_{R}$ , respectively, are the methods of choice at $\nu_{R}=20$ kHz to transfer the magnetization from protons to quadrupolar nuclei. At $\nu_{R}=62.5$ kHz, the RINEPT-CWc- $\mathrm{SR}4_{1}^{2}$ (270 ${}_{0}$ 90 ${}_{180}$ ) and PRESTO- $R16_{7}^{6}$ (270 ${}_{0}$ 90 ${}_{180}$ ) sequences with rf requirements of ca. 4 $\nu_{R}$ and $2.3\nu_{R}$ , respectively, result in the most robust and efficient transfers. At both MAS frequencies, the RINEPT and PRESTO techniques complement each other since the latter is dipolar truncated, whereas the former is not. As a result, the RINEPT sequences must be chosen to observe simultaneously protonated and unprotonated sites, whereas the PRESTO schemes can be employed for the selective observation of quadrupolar nuclei in proximity to protons. These techniques are expected to be useful for transferring the DNP-enhanced magnetization of protons to quadrupolar nuclei in indirect MAS DNP experiments at $\nu_{R}\geq 20$ kHz, notably used at high magnetic fields (Nagashima et al., 2021, 2020; Rankin et al., 2019; Berruyer et al., 2020). We also show that they can be used to correlate the NMR signals of protons and quadrupolar nuclei at high MAS frequencies.

## Supplement

10.5194/mr-2-447-2021-supplement The supplement related to this article is available online at: https://doi.org/10.5194/mr-2-447-2021-supplement.

## Data Availability

The raw data are available on the Zenodo site at https://doi.org/10.5281/zenodo.4896852 (Gómez et al., 2021).
