# Supplementary material for: Improved NMR transfer of magnetization from protons to half-integer spin quadrupolar nuclei at moderate and high magic-angle spinning frequencies
Source: Magn Reson (Gott). 2021 Jun 17;2(1):447–64. doi: 10.5194/mr-2-447-2021 (PMC10539806; doi:10.5194/mr-2-447-2021)
Supplement: The supplement related to this article is available online at: https://doi.org/10.5194/mr-2-447-2021-supplement. [file mr-2-447-supplement.pdf]

Supplement of Magn. Reson., 2, 447–464, 2021
https://doi.org/10.5194/mr-2-447-2021-supplement
© Author(s) 2021. CC BY 4.0 License.

Supplement of

Improved NMR transfer of magnetization from protons to half-integer spin quadrupolar nuclei at moderate and high magic-angle spinning frequencies

Jennifer S. Gómez et al.

Correspondence to: Olivier Lafon (olivier.lafon@univ-lille.fr)
and Jean-Paul Amoureux (jean-paul.amoureux@univ-lille.fr)

The copyright of individual parts of the supplement might differ from the article licence.

Supporting Information

Table S1. Selected RN_n^ν $|m| = 2$ SQ hetero-nuclear dipolar recoupling for ν_R = 20 kHz.

$\mathcal{R}$	RN_n^ν	ϕ°	ν_1/ν_R	κ	$\kappa/\left\Vert\kappa_{\{1,2\}}^{DD_1\times DD_2}\right\Vert_2$	$\kappa/\left\Vert\kappa_{\{1,2\}}^{CSA\times CSA}\right\Vert_2$	$\kappa/\left\Vert\kappa_{\{1,2\}}^{\delta iso\times\delta iso}\right\Vert_2$
180_0	$R22_2^7$	57	5.5	0.178	162	7.12	17.58
	$R28_3^5$	51	4.67	0.176	156	5.08	18.29
	$R18_2^5$	50	4.5	0.175	140	7.20	18.49

Table S2. Selected RN_n^ν $|m| = 2$ SQ hetero-nuclear dipolar recoupling with 45° ≤ ϕ ≤ 135° for ν_R = 62.5 kHz.

$\mathcal{R}$	RN_n^ν	ϕ°	ν_1/ν_R	κ	$\kappa/\left\Vert\kappa_{\{1,2\}}^{DD_1\times DD_2}\right\Vert_2$	$\kappa/\left\Vert\kappa_{\{1,2\}}^{CSA\times CSA}\right\Vert_2$	$\kappa/\left\Vert\kappa_{\{1,2\}}^{\delta iso\times\delta iso}\right\Vert_2$
$90_0240_{90}90_0$	$R10_4^3$	54	2.92	0.227	39.63	2.82	12.63
	$R14_6^5$	64.3	2.72	0.232	36.33	1.87	12.39
	$R12_5^4$	60	2.80	0.230	36.08	2.25	12.47
	$R12_7^8$	120	2.00	0.227	35.96	1.61	7.72
270_090_{180}	$R16_7^6$	67.5	2.28	0.150	17.96	1.85	3.50×10^{10}
	$R16_7^{10}$	112.5	2.28	0.150	17.96	1.85	3.50×10^{10}
	$R14_6^5$	64.3	2.33	0.150	15.90	2.33	3.58×10^{10}
	$R14_6^9$	115.7	2.33	0.150	15.90	2.15	3.58×10^{10}
$90_{-45}90_{45}90_{-45}$	$R10_4^3$	54	1.88	0.186	16.70	2.97	15.07
	$R18_7^5$	50	1.93	0.189	15.73	1.98	25.49
	$R14_6^5$	64.3	1.75	0.177	15.55	2.09	5.49
	$R12_5^4$	60	1.80	0.181	15.17	2.47	8.11
180_0	$R14_6^5$	64.3	1.16	0.085	5.35	2.26	1.34
	$R14_6^9$	115.7	1.16	0.085	5.35	2.26	1.34
	$R16_7^6$	67.5	1.14	0.082	4.90	1.98	1.09
	$R16_7^{10}$	112.5	1.14	0.082	4.90	1.98	1.09

Table S3. Selected RN_n^ν $|m| = 2$ SQ hetero-nuclear dipolar recoupling built from single π pulses with 20° ≤ ϕ ≤ 160° and κ ≥ 0.15 for ν_R = 62.5 kHz.

$\mathcal{R}$	RN_n^{ν}	ϕ /°	ν_1/ν_R	κ	$\kappa/\left\Vert \kappa_{\{1,2\}}^{DD_1 \times DD_2} \right\Vert_2$	$\kappa/\left\Vert \kappa_{\{1,2\}}^{CSA \times CSA} \right\Vert_2$	$\kappa/\left\Vert \kappa_{\{1,2\}}^{\delta iso \times \delta iso} \right\Vert_2$
	$R28_5^4$	25.7	2.75	0.163	24.42	3.34	26.42
180_0	$R22_4^3$	24.5	2.75	0.162	22.84	4.10	27.24
	$R16_3^2$	22.5	2.67	0.161	16.26	5.21	28.89

Table S4. Selected RN_n^{ν} $|m| = 2$ two-spin order hetero-nuclear dipolar recoupling.

$\mathcal{R}$	RN_n^{ν}	ϕ°	ν_1/ν_R	κ	$\kappa/\left\Vert \kappa_{\{1,2\}}^{DD_1 \times DD_2} \right\Vert_2$	$\kappa/\left\Vert \kappa_{\{1,2\}}^{CSA \times CSA} \right\Vert_2$	$\kappa/\left\Vert \kappa_{\{1,2\}}^{\delta iso \times \delta iso} \right\Vert_2$
	$R16_4^9$	101	4.66	0.131	63.17	16.48	9.31
	$R20_5^{11}$	99	4.66	0.131	60.68	16.59	14.45
	$R12_3^7$	105	4.66	0.131	51.25	16.11	9.70
$90_0 240_{90} 90_0$	$R16_4^7$	79	4.66	0.131	45.52	15.76	13.60
	$R28_7^{10}$	64	4.66	0.131	44.55	14.06	11.98
	$R20_5^9$	81	4.66	0.131	44.30	15.95	14.46
	$R12_3^5$	75	4.66	0.131	43.91	15.40	12.83
	$SR4_1^2$	90	4.66	0.131	42.37	22.65	10.48
	$R28_7^{11}$	71	3	0.191	39.81	10.05	6.10
	$R20_5^8$	72	3	0.191	39.74	10.26	5.49
	$R8_2^3$	67.5	3	0.191	39.43	9.42	7.88
$90_{-45} 90_{45} 90_{-45}$	$R8_2^{11}$	67.5	3	0.191	39.43	9.42	7.88
	$R24_6^{10}$	75	3	0.191	39.32	10.66	4.22
	$R28_7^{10}$	64.3	3	0.191	38.82	8.65	10.13
	$R12_3^5$	75	3	0.191	38.33	10.66	4.22
	$SR4_1^2$	90	3	0.191	19.95	19.48	1.33
	$R24_6^{11}$	82.5	4	0.212	33.12	25.46	8.67×10^{10}
	$R20_5^9$	81	4	0.212	31.85	25.19	8.67×10^{10}
$270_0 90_{180}$	$R20_5^{11}$	99	4	0.212	31.85	25.19	8.67×10^{10}
	$R16_4^7$	78.8	4	0.212	28.56	24.69	8.67×10^{10}
	$R16_4^9$	101.2	4	0.212	28.56	24.69	8.67×10^{10}
	$R12_3^5$	75	4	0.212	20.84	23.58	8.67×10^{10}

	$R12_{3}^{7}$	105	4	0.212	20.84	23.58	8.67×10^{10}
	$SR4_{1}^{2}$	90	4	0.212	35.21	149.93	8.67×10^{10}
	$R16_{4}^{7}$	78.8	2	0.25	19.65	10.52	2.78
	$R16_{4}^{9}$	115.7	2	0.25	19.65	10.52	2.78
180_{0}	$R12_{3}^{5}$	75	2	0.25	18.9	9.89	3.74
	$R12_{3}^{7}$	105	2	0.25	18.9	9.89	3.74
	$SR4_{1}^{2}$	90	2	0.25	13.2	22.98	1.56

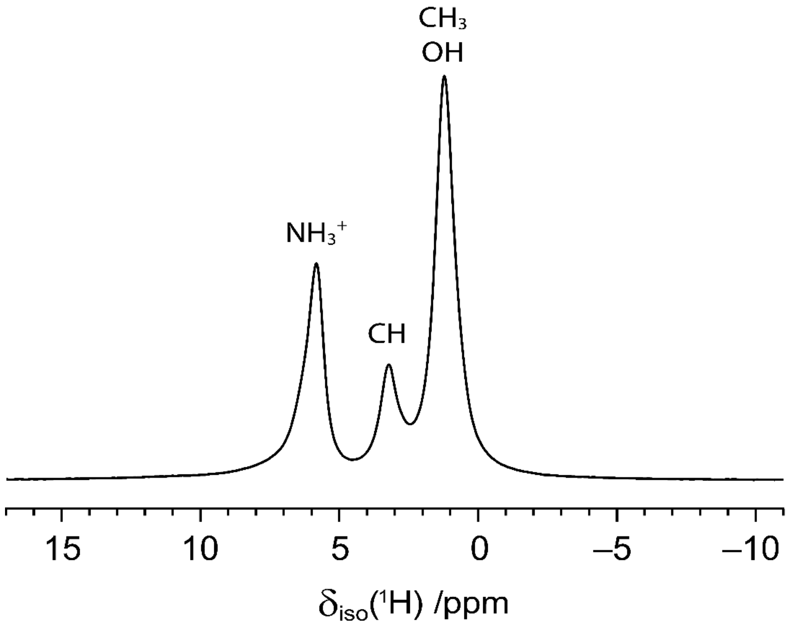

Figure S1: ^{1}H MAS spectrum of $AlPO_4$-14 acquired at B_0 = 18.8 T and ν_R = 20 kHz by averaging 16 transients separated by a recycle interval of 1 s, using the DEPTH pulse sequence for probe background suppression, with $\nu_1 \approx$ 208 kHz (Cory and Ritchey, 1988).

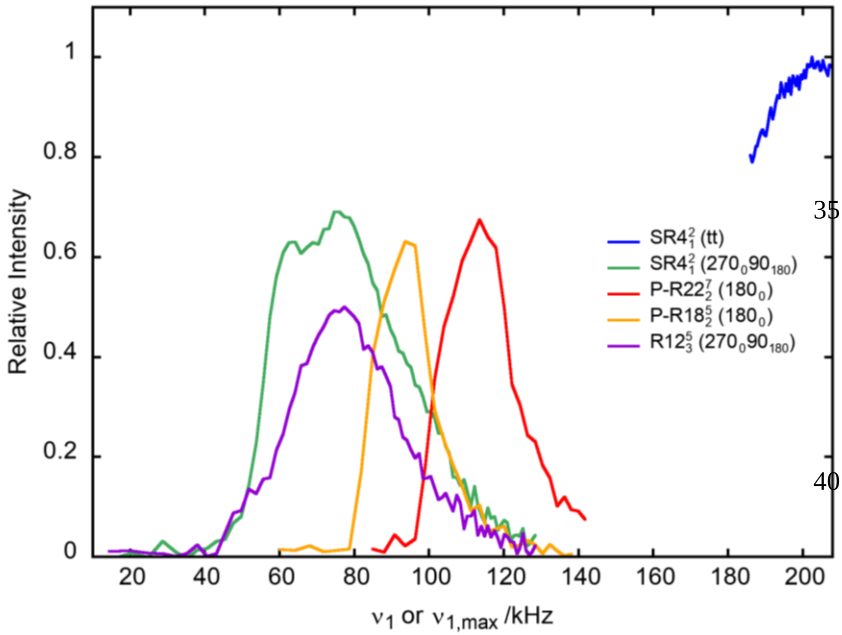

Figure S2: $^{27}AlO_4$ signal of $AlPO_4$-14 at ν_R = 20 kHz as function of ν_1 or $\nu_{1,max}$ of the recoupling for PRESTO-R22$^7_2$(180$_0$) and -R18$^5_2$(180$_0$) as well as RINEPT-CWc-SR4$^2_1$ (tt), -SR4$^2_1$ (270$_0$90$_{180}$) and -R12$^5_3$ (270$_0$90$_{180}$). For each curve, τ was fixed to its optimum value given in Table 2.

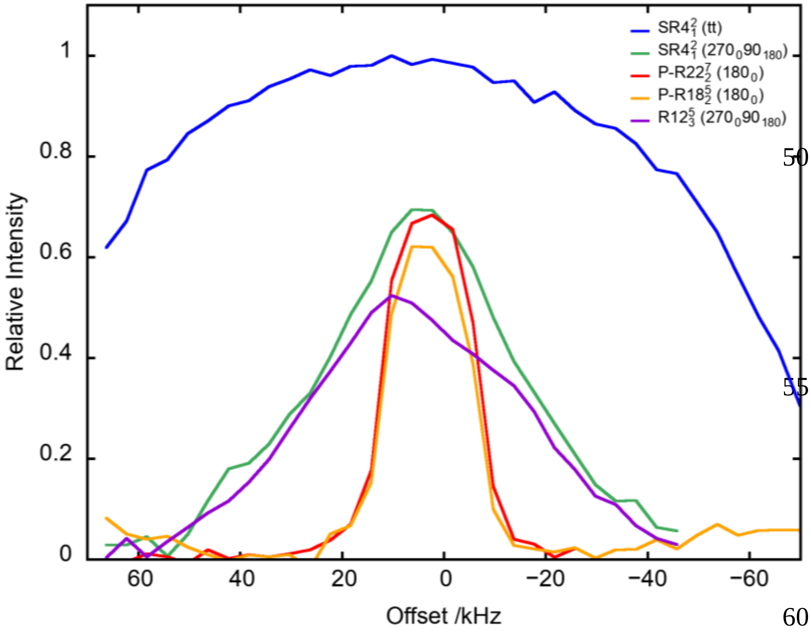

Figure S3: $^{27}AlO_4$ signal of $AlPO_4$-14 at ν_R = 20 kHz as function of offset of the recoupling for PRESTO-R22$^7_2$(180$_0$) and -R18$^5_2$(180$_0$) as well as RINEPT-CWc-SR4$^2_1$ (tt), -SR4$^2_1$ (270$_0$90$_{180}$) and -R12$^5_3$ (270$_0$90$_{180}$). For each curve, τ and ν_1 or $\nu_{1,max}$ were fixed to their optimum values given in Table 2.

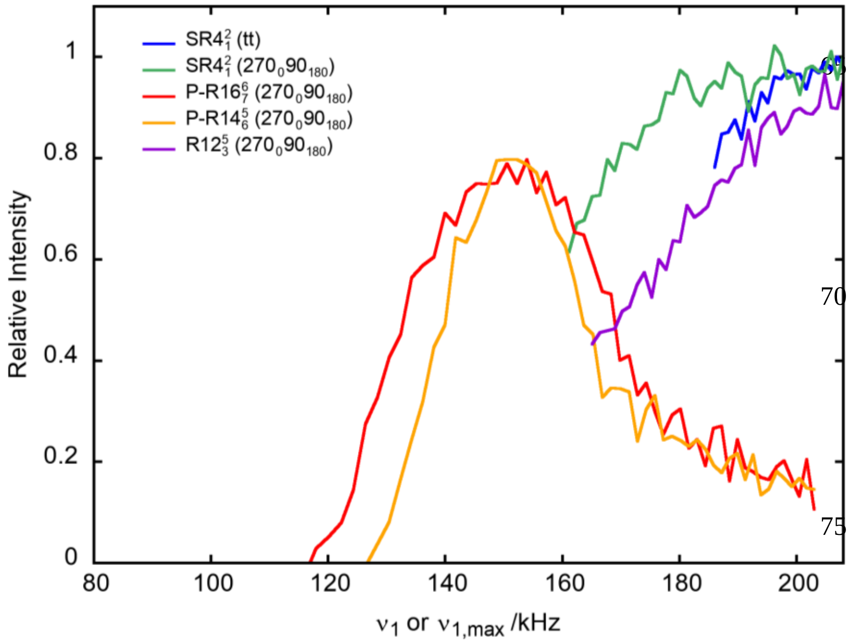

Figure S4: $^{27}AlO_4$ signal of $AlPO_4$-14 at ν_R = 62.5 kHz as function of ν_1 or $\nu_{1,max}$ of the recoupling for PRESTO-R16_7^6($270_0 90_{180}$) and -R14_6^5($270_0 90_{180}$) as well as RINEPT-CWc-*S*R4_1^2 (tt), -*S*R4_1^2 ($270_0 90_{180}$) and -R12_3^5 ($270_0 90_{180}$). For each curve, τ was fixed to its optimum value given in Table 4.

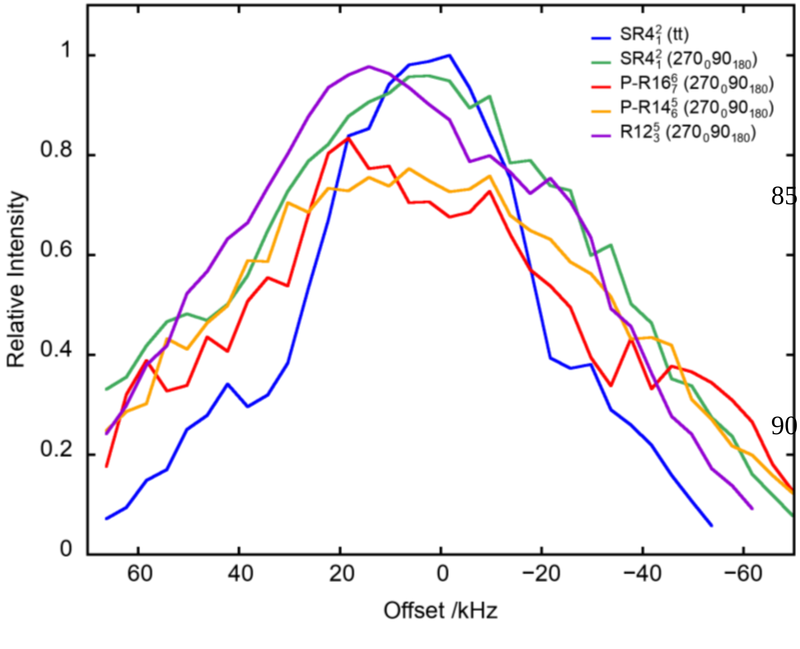

Figure S5: $^{27}AlO_4$ signal of $AlPO_4$-14 at ν_R = 62.5 kHz as function of offset of the recoupling for PRESTO-R16_7^6($270_0 90_{180}$) and -R14_6^5($270_0 90_{180}$) as well as RINEPT-CWc-*S*R4_1^2 (tt), -*S*R4_1^2 ($270_0 90_{180}$) and -R12_3^5 ($270_0 90_{180}$). For each curve, τ and ν_1 or $\nu_{1,max}$ were fixed to their optimum values given in Table 4.

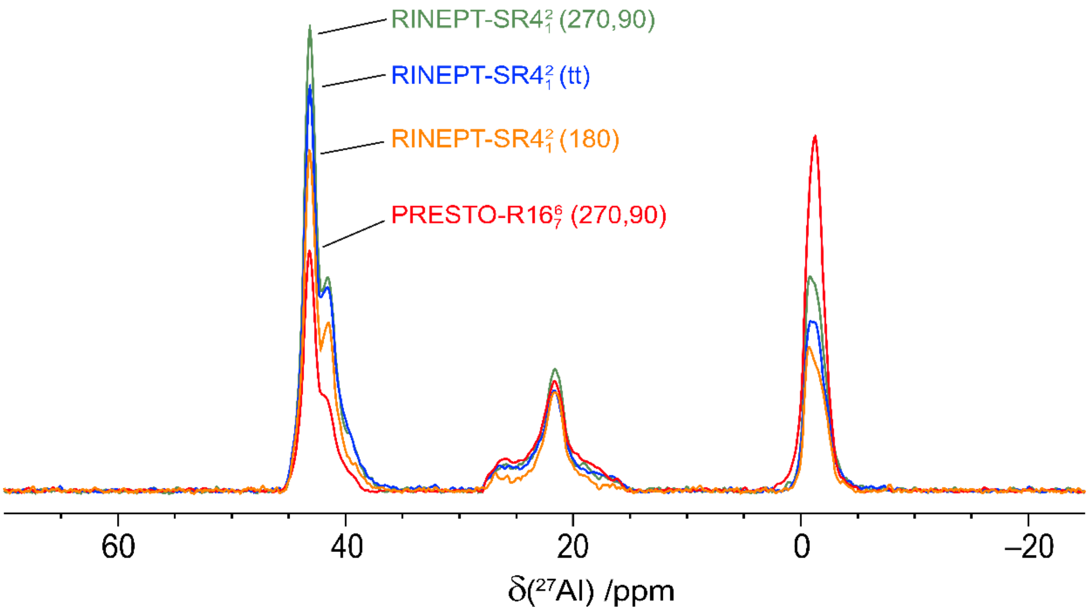

Figure S6: Skyline projections along F_2 of ^1H-^{27}Al HETCOR 2D spectra of $AlPO_4$-14 recorded with RINEPT-CWc-SR4_1^2($270_0 90_{180}$), SR4_1^2(tt), SR4_1^2($180_0 90_{180}$) and PRESTO-R16_7^6 ($270_0 90_{180}$) transfers. All 2D spectra were acquired using NUS 25% in 72 min.

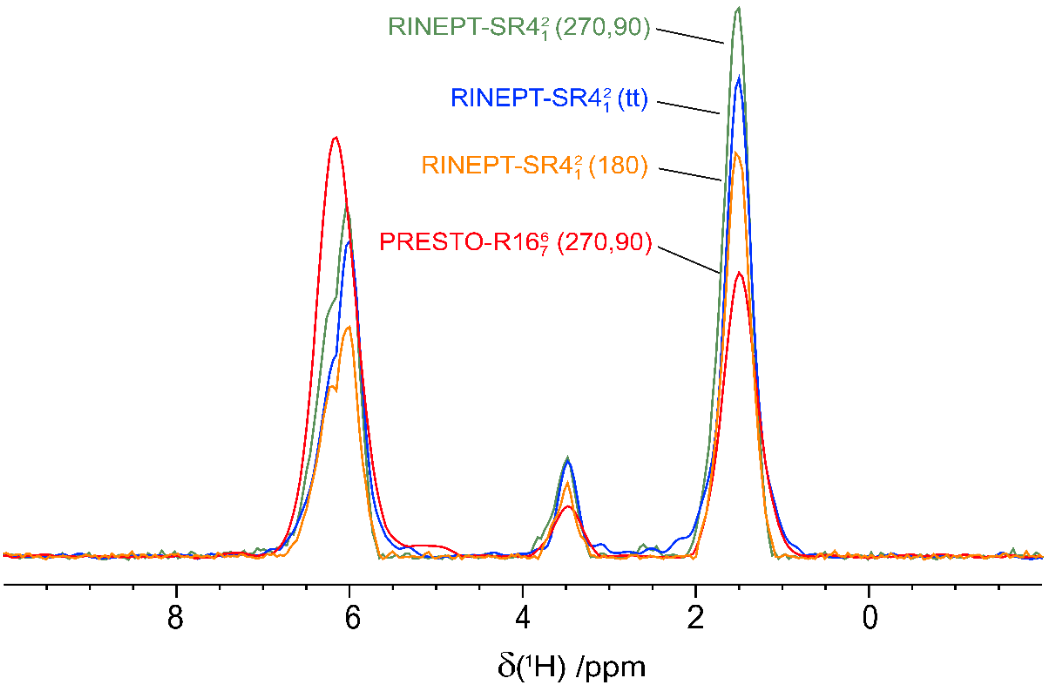

Figure S7: Skyline projections along F_1 of ^1H-^{27}Al HETCOR 2D spectra of $AlPO_4$-14 recorded with RINEPT-CWc-SR4_1^2($270_0 90_{180}$), SR4_1^2(tt), SR4_1^2($180_0 90_{180}$) and PRESTO-R16_7^6 ($270_0 90_{180}$) transfers. All 2D spectra were acquired using NUS 25% in 72 min.

Table S5. Distances between the different hydrogen atoms and their closest Al neighbours in the structure of isopropylamine templated $AlPO_4$-14 determined from X-ray diffraction. (Broach et al., 2003) The H and Al atoms are numbered according to the cif file.

H	Al	r_{HAl} /Å
H1 (OH)	$Al4O_6$	2.496
	$Al4O_6$	2.499
	$Al1O_5$	2.503
	$Al2O_4$	4.299
H2 (NH_3)	$Al4O_6$	3.069
	$Al2O_4$	3.779
H3 (NH_3)	$Al3O_4$	3.778
	$Al4O_6$	3.960
H4 (NH_3)	$Al2O_4$	3.479
	$Al1O_5$	3.801
H5 (CH)	$Al2O_4$	3.737
	$Al1O_5$	4.850
H6 $(CH_3)_1$	$Al1O_5$	3.655
	$Al3O_4$	4.594
H7 $(CH_3)_1$	$Al3O_4$	4.082
	$Al1O_5$	4.320
H8 $(CH_3)_1$	$Al2O_4$	3.772
	$Al3O_4$	4.651
H9 $(CH_3)_2$	$Al4O_6$	3.888
	$Al3O_4$	4.124
H10 $(CH_3)_2$	$Al4O_6$	3.509
	$Al3O_4$	4.502
H11 $(CH_3)_2$	$Al4O_6$	3.970
	$Al3O_4$	4.048

Broach, R. W., Wilson, S. T., Kirchner, R. M.: Corrected crystallographic tables and figure for as-synthesized $AlPO_4$-14, Microporous and Mesoporous Materials, 57, 211–214, https://doi.org/10.1016/S1387-1811(02)00563-2, 2003.

Cory, D. G. and Ritchey, W. M.: Suppression of signals from the probe in bloch decay spectra, 80, 128–132, https://doi.org/10.1016/0022-2364(88)90064-9, 1988.

Pulse sequence for *D*-RINEPT using $SR4_1^2$ ($270_0 90_{180}$) or $R12_3^5$ ($270_0 90_{180}$) recouplings

```
;INEPT for non-selective polarization transfer
;with decoupling during acquisition
; made of 2 pulses
; different recoupling sequences and composite pulses available
; modified by Julien Trébosc and Jennifer Gómez (2020)
; AVANCE NEO

;d0 initial t1 evolution time (=0)
;d6 probe dead time (should be D6=DE)
;d7 RF offset delay
;d5 Delay after last recoupling for Tr/2
;d8 Delay after last recoupling for Tr/4
;pl1 p1 and p2 power level
;pl12 Heteronuclear dipolar decoupling
;pl19 Presat pulse
;pl2 not used
;pl20 Presat pulse
;pl21 p3 and p4 power level
;pl22 initial spin lock
;pl33 CW23 decoupling
;pl43 CW45 decoupling
;pl44 CW67 decoupling
;pl11 dipolar recoupling power (sr4/sfam)
;spnam5 dipolar recoupling shape pulse
;sp5 power for recoupling shape
;p16 : requested recoupling time
;p17 : actual recoupling time
;l11 sr4/sfam repetition
;cnst30: Tanh/tan offset
;cnst31: spinning speed in Hz
;cnst3: Tanh/tan shape pulse step (ns)
;p1 90 degree pulse for X
;p2 180 degree pulse for X
;p3 90 degree pulse for 1H
;p4 180 degree pulse for 1H
;p6 pulse of the recoupling sequence
;p19 presat pulse for 1H
;p20 presat pulse for X
;p22 initial spin lock for Tr/2
;p23 initial spin lock for Tr/4
;p33 CW45 decoupling for Tr/2
;p34 CW45 decoupling for Tr/4
;p43 CW23 decoupling for Tr/2
;p44 CW67 decoupling for Tr/2
```

```
;p45 CW23 decoupling for Tr/4
;p46 CW67 decoupling for Tr/4

;d1 : relaxation delay; 1-5 * T1
;NS: 16 * n, total number of scans: NS * TD0
;DS: 16
;cpd1: decoupling during R3
;cpdprg1: decoupling during R3
;cpd2: decoupling during AQ and t1
;cpdprg2: decoupling during AQ and t1
;cpd3: decoupling during AQ
;cpdprg3: decoupling during AQ


#include <Avance.incl>

; storeVC option to store VClist used when popting MAS
#ifdef storeVC
#define VCstored vclab, 1u \n lo to vclab times c
#else
#define VCstored
#endif

;-)))))))
#include "presat.incl"
;-)
#ifndef PRESATf2
#undef PRESAT2
#define PRESAT2(f2)
#endif
;-)
#ifndef PRESATf1
#undef PRESAT1
#define PRESAT1(f1)
#endif
;-(((((((
;----------- DECOUPLING ----------------
#include "decouple.incl"

#ifdef decF2
#define decF2off do:f2
#define decF2aqon cpds2:f2
#else
#define decF2aqon
#define decF2off
#endif

define delay RF
define delay dummy
```

```
#ifdef _SR4_cp1
;this is SR4 sequence using composite pulse 270(0)-90(180)
#define phaseRN (360) {{{90 270   270 90}*2}^180}^120^240
"p6=0.25s/cnst31"
"p7=p6*3/4.0" ; p270 deg
"p8=p6/4.0"   ; p90 deg
; we have p6 = p7 + p8
;"l11=trunc((p16/p6)/4+0.5)"   ; +0.5 will round to nearest integer
"p17=2*p6*2*l11"
"RF=250e3/p8"
"dummy=RF+p17"
#endif

#ifdef _R1235_cp1
;this is R12_3^5 sequence using composite pulse 270(0)-90(180)
#define phaseRN (360)  75 255   285 105
"p6=0.25s/cnst31"
"p7=p6*3/4.0" ; p270 deg
"p8=p6/4.0"   ; p90 deg
; we have p6 = p7 + p8
;"l11=trunc((p16/p6)/4+0.5)"   ; +0.5 will round to nearest integer
"p17=2*p6*2*l11"
"RF=250e3/p8"
"dummy=RF+p17"
#endif

;............................................
"d24=p3"
"p2=p1*2"
"p4=p3*2"
;"d6=de"
"p22=0.5s/(cnst31)-p3/2.0"
"p23=0.25s/(cnst31)-p3/2.0"
"d5=0.5s/(cnst31)-d6"
"d8=0.25s/(cnst31)-d6"
"p33=0.5s/(cnst31)-p3"
"p34=0.25s/(cnst31)-p3-p4"
"p44=0.5s/(cnst31)-p4/2.0"
"p46=0.25s/(cnst31)-p4/2.0"
"p55=0.5s/(cnst31)-d6"
"p43=0.5s/(cnst31)-p4/2.0-p3"
"p45=0.25s/(cnst31)-p4/2.0"
"d7=0.00000005s"
"plw43=plw33"
"plw44=plw33"

"in0=inf1"

define delay showInAsed
"showInAsed=cnst3+dummy"
```

```
1 ze
VCstored
"showInAsed=cnst3+dummy"

2 30m decF2off
  PRESAT2(f2)
  d1 rpp16 rpp17 rpp14 rpp15 ; not necessary to use different phases and reset but...
  PRESAT1(f1)
  (10u pl21):f2 (10u pl1 ph2):f1
  (p3 ph1):f2


#ifdef _iSL
if "l11 % 2 == 0"
 {
  (p22 pl22 ph27):f2
 }
else
 {
  (p23 pl22 ph27):f2
 }
#endif

  d0

sr4_1, (p7 pl11 ph16^):f2
       (p8 pl11 ph16^):f2
       (p7 pl11 ph16^):f2
       (p8 pl11 ph16^):f2
  lo to sr4_1 times l11

if "l11 % 2 == 0"
 {
  (center (p3 pl21 ph18 p43 pl43 ph21 p4 pl21 ph2 p43 pl43 ph22 p3 pl21 ph18):f2 (p2 ph11):f1 )
 }
else
 {
  (center (p45 pl43 ph18 p4 pl21 ph2 p45 pl43 ph18):f2 (p2 ph11):f1 )
 }

sr4_2, (p7 pl11 ph17^):f2
       (p8 pl11 ph17^):f2
       (p7 pl11 ph17^):f2
       (p8 pl11 ph17^):f2
  lo to sr4_2 times l11

if "l11%2 == 0"
 {
  (center (p3 pl21 ph18 p33 pl33 ph23 p33 pl33 ph24 p3 pl21 ph3):f2 (p1 ph12):f1 )
```

```
 }
else
 {
  (center (p4 pl21 ph5 p3 pl21 ph3 p34 pl33 ph21 p34 pl33 ph22 p4 pl21 ph5 p3 pl21 ph3):f2 (p1 ph12):f1 )
 }

sr4_3, (p7 pl11 ph15^):f2
       (p8 pl11 ph15^):f2
       (p7 pl11 ph15^):f2
       (p8 pl11 ph15^):f2
  lo to sr4_3 times l11

if "l11%2 == 0"
 {
  (center (p44 pl44 ph25 p4 pl21 ph2 p44 pl44 ph26):f2 (p2 ph13):f1 )
 }
else
 {
  (center (p46 pl44 ph25 p4 pl21 ph2 p46 pl44 ph26):f2 (p2 ph13):f1 )
 }

sr4_4, (p7 pl11 ph14^):f2
       (p8 pl11 ph14^):f2
       (p7 pl11 ph14^):f2
       (p8 pl11 ph14^):f2
  lo to sr4_4 times l11

if "l11%2 == 0"
 {
  d5 decF2aqon
 }
else
 {
  d8 decF2aqon
 }
  go=2 ph31
  10u decF2off
  30m mc #0 to 2 F1PH(ip1,id0)

HaltAQ, 1m

exit

ph0=0
ph2=0
ph3=0
ph4=0
ph5= (360) 45
ph6=0
ph7=0
```

```
ph10=0
ph11={{0}*2}^2
ph12={{0}*4}^2
ph13={{0}*8}^2^1^3
ph18=1
ph21=0
ph22=2
ph23=0
ph24=2
ph25=0
ph26=2
ph27=0 2
ph28=0
ph29=3
ph16= phaseRN
ph17= phaseRN
ph15= phaseRN
ph14= phaseRN

#ifdef opt1D
ph1=1 3 0 2
ph31=3 1 2 0
#else
ph1=1 3
ph31={{{1 3}^0}^2}^0^2^2
#endif
presatPH
```

SIMPSON input file for *D*-RINEPT-CWc-$SR4_1^2$(tt)

```
spinsys {
  channels   1H 13C
  nuclei     1H 13C 1H 1H 1H
# single pair
  shift      1 0 6000 0 0 30 0
  dipole     1 2 -2575 0 0 0
# 2 1H
  shift      3 0 6000 0 0 30 0
  dipole     3 2 0 0 109 0
  dipole     1 3 -7000 0 109 0
# 3 1H
  shift      4 0 6000 0 0 30 0
  dipole     4 2 0 0 109 120
  dipole     1 4 -7000 0 109 120
  dipole     3 4 -7000 0 90 30
# 4 1H
  shift      5 0 6000 0 0 30 0
  dipole     5 2 0 0 109 240
  dipole     1 5 -7000 0 109 240
```

```
  dipole     3 5 -7000 0 90 90
  dipole     4 5 -7000 0 90 330
}

par {
  proton_frequency 400e6
  spin_rate        12500
  sw               spin_rate/2.0
  np               30
  crystal_file     rep66
  gamma_angles     7
  start_operator   I1z
  detect_operator  I2p
  verbose          1101
  variable HRF     100000
  variable DRF     92000
  variable CRF     100000
variable RFmax   spin_rate*11
  variable offmax  2000000
  variable I       1.0/2.0
}

proc gen_tanhtan_shape {pulse_length steps offmax xi K} {
# generate a tanhtan shape with given :
# pulse_length : length of ulse in us
# steps : number of steps defining the shape
# offset : maximum frequency offset of tanhtan sweep
# xi : tanhtan xi parameter
# K : tanhtan kappa parameter
set nhalf [expr $steps/2]
set amp_list [list ]
set phase_list [list ]
for {set i 0} {$i < $steps} {incr i} {
    set x [expr 1.0*$i/(1.0*$steps)]
    if {$i<$nhalf} {
        lappend amp_list [expr tanh(2*$xi*$x)]
      } else {
        lappend amp_list [expr tanh(2*$xi*(1-$x))]
      }
            lappend phase_list [expr -360*$offmax*$pulse_length*(1e-6)*log(abs(cos($K*(1-2*$x))))/(2*tan($K)*$K)]
}
set Tinc [expr 1.0*$pulse_length/$steps]
return [list $amp_list $phase_list $Tinc]
}

proc tanhtan_pulse {shape RF phase } {
# generate simpson pulse following shape argument containing amplitude and phase lists
# shape: as generated by gen_tanhtan_shape procedure
# RF : global maximum RFfield of shape
# phase : global phase of shape
```

```
  set amp_list [lindex $shape 0]
  set phase_list [lindex $shape 1]
  set Tinc [lindex $shape 2]
  foreach amp $amp_list phi $phase_list {
    pulse $Tinc [expr $amp*$RF] [expr $phase+$phi] 0 0
  }
}

proc pulseq {} {
  global par
  maxdt 6.0

  set H90  [expr 0.25e6/$par(HRF)]
  set H180 [expr 0.50e6/$par(HRF)]
  set C90   [expr 0.25e6/$par(CRF)]
  set C180  [expr 0.50e6/$par(CRF)]
  set Taur  [expr 1.0e6/$par(spin_rate)]
   set Td90  [expr 0.5e6/$par(spin_rate)-$H90/2]
   set Td180  [expr 0.5e6/$par(spin_rate)-$H180/2]
  # RN_n^nu parameters
  set N        4.
  set nu    2.
   # set n     1.
    set php  [expr 180*$nu/$N]
    set S90 [expr 0.25e6/$par(RFmax)]
    set S180 [expr 0.50e6/$par(RFmax)]


   set n    100
   set Tp [expr 0.25*$Taur]
   set Tpd [expr 0.25*$Taur]
   # set Q    7.7
   set xi  10.0
   set K    atan(20)
   set pi   [expr atan(1)*4]

   set shape [gen_tanhtan_shape $Tp $n $par(offmax) $xi $K ]

  set ph1 0
  set ph2 120
  set ph3 240

# SR4 using tanhtan inversion
# full block with supercycling
 set superCycling {0 180 120 300 240 60}
 reset
 foreach ph1 $superCycling  {
 reset
 for {set s 0 } {$s<$N/2} {incr s } {
        delay [expr $Tpd/2-$Tp/2]
```

```
      tanhtan_pulse $shape $par(RFmax) [expr $php+$ph1]
         delay [expr $Tpd/2-$Tp/2]
         delay [expr $Tpd/2-$Tp/2]
      tanhtan_pulse  $shape $par(RFmax) [expr -$php+$ph1]
         delay [expr $Tpd/2-$Tp/2]
   }
   store $ph1
   }

   reset [expr -$H90]
   pulse $H90 $par(HRF) 90 0 0
  # pulse $Td90 $par(DRF) 0 0 0
   store 19

   reset
   pulse $Td180 $par(DRF) 0 0 0
   pulse $H180 $par(HRF) 0 $par(CRF) 0
   pulse $Td180 $par(DRF) 180 0 0
   store 20

     reset
   pulse $Td90 $par(DRF) 0 0 0
   pulse $H90 $par(HRF) 0 $par(CRF) 0
   pulse $Td90 $par(DRF) 180 0 0
   store 21

     reset
   pulse $Td180 $par(DRF) 0 0 0
   pulse $H180 $par(HRF) 0 $par(CRF) 0
   pulse $Td180 $par(DRF) 180 0 0
   store 22


   reset
   # prop [expr (0%[llength $superCycling])*[lindex $superCycling 1]]
       prop [lindex $superCycling 0]
   store 10

   for {set i 0} {$i < $par(np)} {incr i} {
 #reset
   reset [expr -$H90]
    # pulseid $H90 $par(HRF) 90 0 0
   prop 19
   prop 10
   prop 20
   prop 10
   prop 21
   prop 10
   prop 22
   prop 10
```

```
  pulse [expr $Taur/2.0] $par(DRF) 0 0 0
  acq

   reset
   prop 10
#   puts [expr (($i+1)%[llength $superCycling])*[lindex $superCycling 1]]
 #  prop [expr (($i+1)%[llength $superCycling])*[lindex $superCycling 1]]
   prop [lindex $superCycling [expr (($i+1)%6)]]
   store 10

  }
}

proc main {} {
  global par

  set FileRe [open "$par(name)-Re.res" w]
  set FileIm [open "$par(name)-Im.res" w]
  set FileAbs [open "$par(name)-Abs.res" w]

  set f [fsimpson]
  set c 0
  for {set i 1} {$i <= $par(np)} {incr i} {
    incr c
    set Sr [findex $f $c -re]
    set Si [findex $f $c -im]
    puts $FileRe "[expr 1.0e3*$i/$par(sw)] [expr $Sr]"
    puts $FileIm "[expr 1.0e3*$i/$par(sw)] [expr $Si]"
    puts $FileAbs "[expr 1.0e3*$i/$par(sw)] [expr sqrt($Sr**2+$Si**2)]"
   }
  funload $f
  close $FileRe
  close $FileIm
  close $FileAbs
}
```
